# *Fusobacterium nucleatum-*derived succinic acid aggravates colitis by triggering macrophage pro-inflammatory phenotypic transformation via SUCNR1/NF-κB axis

**DOI:** 10.1080/19490976.2026.2702183

**Published:** 2026-07-15

**Authors:** Suqi Zeng, Shanshan Jiang, Jianxuan Sun, Yan Chen, Meiqi Qiu, Jiao Li, Qingzhi Lan, Yu Pu, Zongbiao Tan, Xingzhou Guo, Haodong He, Shuo Wang, Luyun Zhang, Yafei Liu, Jiaming Hu, Ruxue Wang, Fei Liao, Jixiang Zhang, Weiguo Dong

**Affiliations:** a Department of Gastroenterology, Renmin Hospital of Wuhan University, Wuhan University, Wuhan, People's Republic of China; b Key Laboratory of Hubei Province for Digestive System Disease, Wuhan, Hubei, People's Republic of China; c Central Laboratory, Renmin Hospital of Wuhan University, Wuhan, Hubei, People's Republic of China; d Division of Gastroenterology and Hepatology, Shanghai Institute of Digestive Disease, NHC Key Laboratory of Digestive Diseases, State Key Laboratory of Systems Medicine for Cancer, Renji Hospital, School of Medicine, Shanghai Jiao Tong University, Shanghai, People's Republic of China; e Department of Urology, Tongji Hospital, Tongji Medical College, Huazhong University of Science and Technology, Wuhan, People's Republic of China; f Chengdu Second People's Hospital Affiliated to Sichuan University, Chengdu, People's Republic of China; g Department of Pathology, Renmin Hospital of Wuhan University, Wuhan University, Wuhan, People's Republic of China

**Keywords:** Inflammatory bowel disease, *Fusobacterium nucleatum*, succinic acid, SUCNR1, macrophage, NF-κB

## Abstract

*Fusobacterium nucleatum* (*F. nucleatum*) has been increasingly implicated in the pathogenesis of inflammatory bowel disease (IBD), yet the mechanisms underlying its effects remain incompletely defined. In this study, we integrated human fecal and mucosal samples, comparative metabolomics, multiple experimental colitis models, bacterial genetic manipulation, macrophage functional assays, and host signaling analyses to identify a macrophage-centered mechanism through which *F. nucleatum* exacerbates colitis. We show that *F. nucleatum* colonization increases intestinal and systemic levels of its metabolite succinic acid, upregulates the expression of its cognate receptor SUCNR1 on intestinal macrophages, activates NF-κB signaling, and promotes pro-inflammatory macrophage activation. This macrophage inflammatory response is associated with epithelial barrier disruption, increased epithelial apoptosis, and aggravated mucosal and systemic inflammation. A fumarate reductase-deficient (frdA-KO) *F. nucleatum* strain with impaired succinic acid production showed a markedly reduced capacity to activate macrophage NF-κB signaling, induce macrophage inflammatory activation, and aggravate colitis, whereas exogenous succinic acid restored these effects in the *frdA*-KO setting. Moreover, siSUCNR1 and pharmacological NF-κB inhibition substantially attenuated succinic acid-induced macrophage inflammatory activation, supporting the involvement of a SUCNR1–NF-κB signaling cascade. Collectively, these findings demonstrate that *F. nucleatum* exacerbates colitis by producing succinic acid and engaging SUCNR1–NF-κB–dependent inflammatory activation of macrophages, highlighting the *F. nucleatum*–succinic acid–SUCNR1–NF-κB axis as a potential therapeutic target in IBD.

## Introduction

Inflammatory bowel disease (IBD), encompassing Crohn’s disease (CD) and ulcerative colitis (UC), is a chronic relapsing inflammatory condition that imposes significant global health and socioeconomic burdens.^1^ ^,^ ^2^ Despite extensive research, its etiology remains unclear, with contributing factors such as genetic susceptibility, environmental influences, microbial dysbiosis, immune dysregulation, and impaired barrier function.^1^ Recent studies have increasingly focused on the complex interactions between gut microbiota and the host.

*Fusobacterium nucleatum*(*F. nucleatum*) , an obligate anaerobe commonly found in the oral cavity and gastrointestinal tract,^3^ has been shown to be elevated in patients with IBD and in mice with DSS-induced colitis.^4^ Beyond this association, accumulating evidence indicates that *F. nucleatum* can promote intestinal inflammation by impairing epithelial barrier integrity, inducing mucosal immune activation, reshaping the gut microbial ecosystem, and altering local metabolic profiles.^5^ Previous studies have also reported that *F. nucleatum* aggravates experimental colitis through mechanisms involving epithelial barrier disruption, macrophage inflammatory polarization, AKT2 signaling, pyroptosis, and epithelial STAT3 activation.^6^ While these findings support a pathogenic role for *F. nucleatum* in intestinal inflammation, the bacterial effector molecules and host signaling pathways that connect *F. nucleatum* metabolism to macrophage-driven inflammation in IBD remain incompletely defined.

Microbiota-derived metabolites play a key role in host-microbe interactions,^7^ ^,^ ^8^ and several metabolites produced by *F. nucleatum*, including succinic acid, formate, and propionate, have been identified.^9^ Of these, succinic acid is of particular interest because it accumulates to abnormally high levels in individuals with IBD.^10^ ^,^ ^11^ A growing body of work has linked succinic acid to intestinal inflammation. Succinic acid can act as both a metabolic intermediate and an extracellular immunometabolic signal, particularly through succinate receptor 1 (SUCNR1), and has been implicated in macrophage activation, epithelial responses, intestinal inflammation, and fibrosis.^12^ ^,^ ^13^ However, the role of succinic acid in colitis appears context-dependent, with studies reporting both pro-inflammatory and regulatory effects depending on the disease model, cellular target, and inflammatory microenvironment.^14^ Therefore, although succinic acid has been extensively studied in colitis, it remains unclear whether succinic acid specifically derived from *F. nucleatum* functions as a bacterial effector that drives macrophage-dependent intestinal inflammation.

Macrophages are central regulators of intestinal inflammation and exhibit substantial functional heterogeneity in response to microbial, metabolic, and inflammatory cues. Rather than existing as fixed M1 or M2 populations, intestinal macrophages encompass diverse tissue-resident and monocyte-derived subsets with context-dependent inflammatory or reparative functions.^15^ Pro-inflammatory macrophage programs contribute to cytokine production, epithelial injury, and tissue damage, whereas regulatory and repair-associated macrophage states are involved in inflammation resolution and mucosal healing.^16-18^ Thus, targeting macrophage functional reprogramming has emerged as a promising therapeutic approach for IBD.

In this study, we demonstrate that *F. nucleatum* exacerbates experimental colitis by promoting macrophage inflammatory activation through its metabolite succinic acid. Using a fumarate reductase-deficient (frdA-KO) *F. nucleatum* strain with impaired succinic acid production, together with metabolite rescue experiments and functional assays targeting SUCNR1, we identify succinic acid as a key effector mediating *F. nucleatum*-induced macrophage inflammatory responses. Furthermore, we show that the *F. nucleatum*-succinic acid-SUCNR1 axis activates NF-κB signaling in macrophages and is associated with epithelial barrier disruption, increased epithelial apoptosis, and amplified colonic and systemic inflammation. These findings distinguish our work from prior studies that primarily described succinic acid accumulation, SUCNR1 involvement, epithelial injury, or global microbial dysmetabolism in colitis, and define a bacterial metabolite-receptor-signaling axis through which *F. nucleatum* promotes macrophage-driven intestinal inflammation.

## Materials and methods

### Clinical samples

Clinical samples were collected from Wuhan University Renmin Hospital (Wuhan, China) and Renji Hospital, Shanghai Jiao Tong University School of Medicine (Shanghai, China) for complementary microbiota profiling and *F. nucleatum*-targeted analyzes. Different human sample sets were used for distinct analyzes according to sample availability and assay compatibility. Fecal samples were used for high-throughput 16S ribosomal RNA gene sequencing, fresh mucosal biopsies were used for mucosa-associated microbiota profiling and *F. nucleatum*-specific quantitative PCR, and formalin-fixed paraffin-embedded intestinal tissues were used for fluorescence in situ hybridization (FISH).

Fecal samples were collected from two independent cohorts, including a Renmin cohort with healthy controls (CON, *n* = 17), UC patients (*n* = 15), and CD patients (*n* = 15), and a Renji cohort with CON (*n* = 17), UC patients (*n* = 12), and CD patients (*n* = 17). Fresh fecal samples were collected before bowel preparation and0 stored at −80 °C until DNA extraction and sequencing. Detailed demographic and clinical characteristics of participants included in the fecal microbiota analysis are provided in Table S1 and S2.

Fresh intestinal mucosal biopsies were obtained from patients at Wuhan University Renmin Hospital. For mucosa-associated microbiota profiling, biopsies from endoscopically inflamed mucosa were collected from patients with CD (*n* = 6) and UC (*n* = 11) and subjected to high-throughput 16S ribosomal RNA gene sequencing. Detailed clinical characteristics and sampling-site information for participants included in the mucosal microbiota analysis are provided in Table S3. For *F. nucleatum*-specific quantitative PCR analysis, an independent set of mucosal biopsies was collected from IBD patients (*n* = 23) and normal controls (NC, *n* = 14). IBD biopsies were obtained from inflamed mucosal regions. Fresh biopsy specimens were immediately snap-frozen in liquid nitrogen and stored at −80 °C until further analysis. Detailed clinical characteristics and sampling-site information for participants included in the mucosal *F. nucleatum* qPCR analysis are provided in Table S4.

Disease activity was assessed using the Harvey-Bradshaw Index for CD^19^ and the Mayo Score for UC.^20^ For CD, disease activity was categorized according to the Harvey-Bradshaw Index as remission (<5), mild activity (5–7), moderate activity (8–16), and severe activity (>16). For UC, disease activity was categorized according to the Mayo score as mild (3–5), moderate (6–10), and severe (11–12).^20^ ^,^ ^21^ Refractory disease was defined as persistent clinical and/or endoscopic activity despite prior or ongoing treatment with conventional therapy, immunomodulators, or biologic agents, as documented in the medical records. Perianal disease was defined as the presence of a perianal fistula, abscess, or fissure documented at the time of sample collection.

In addition, paraffin-embedded intestinal tissues from IBD patients (*n* = 101) and NC (*n* = 10) were retrieved from the Department of Pathology and subjected to FISH assays. Clinicopathological data were extracted from medical records. Detailed clinicopathological characteristics and sampling-site information for the FISH cohort are provided in Table S5.

Patients with IBD were diagnosed based on clinical, endoscopic, radiological, and histopathological criteria. NC were individuals undergoing routine health examinations or colonoscopy for colorectal cancer screening, with no history of gastrointestinal disorders, gastrointestinal malignancy, or active gastrointestinal inflammation. Patients with infectious diarrhea, emergency gastrointestinal conditions, primary sclerosing cholangitis, pregnancy, recent malignancy, sulfate-containing bowel preparation before endoscopic sampling, prior resection of the ileocecal valve, or a history of antibiotic drug use, probiotics/prebiotics use, or fecal microbiota transplantation within the previous three months were excluded from microbiota-related analyzes.

All participants provided written informed consent, and ethics approval was obtained from Renmin Hospital of Wuhan University (WDRY2025-K115) and Renji Hospital, Shanghai Jiao Tong University School of Medicine (KY2025-128-C).

### High-throughput 16S rRNA gene sequencing

Total genomic DNA was extracted from fecal and mucosal biopsy samples using the TGuide S96 Magnetic Soil/Stool DNA Kit (Tiangen Biotech, Beijing, China) according to manufacturer’s instructions. The V3-V4 hypervariable region of the bacterial 16S rRNA gene was amplified using the primer pairs 338F (5'- ACTCCTACGGGAGGCAGCA-3') and 806R (5'- GGACTACHVGGGTWTCTAAT-3'). PCR products were checked on agarose gel and purified through the Omega DNA purification kit (Omega Inc., Norcross, GA, USA). The purified amplicons were pooled in equimolar amounts, and paired end sequencing (2 × 250 bp) was performed on the Illumina NovaSeq 6000 platform.

### Bioinformatic analysis

High-quality sequences were clustered into operational taxonomic units (OTUs) at 97% sequence similarity using USEARCH (version 10.0). Taxonomy annotation of representative OTU sequences was performed based on the Naive Bayes classifier in QIIME2^22^ using the SILVA database^23^ (release 138.1), with a confidence threshold of 70%. Alpha diversity analysis was performed to identify the complexity of species diversity of each sample utilizing QIIME2 software. Beta diversity was assessed using distance-based metrics and visualized by principal coordinate analysis (PCoA). One-way analysis of variance was used to compare bacterial abundance and diversity. Linear discriminant analysis (LDA) coupled with effect size (LEfSe) was applied to evaluate the differentially abundant taxa.

### Detection of *F. nucleatum*

The primer sequences for *F. nucleatum* have been previously described.^24^ Genomic DNA was extracted from colon tissue using the TIANamp Bacterial DNA Kit (Tiangen Biotech, Beijing, China), following the manufacturer's protocol. DNA concentrations were measured using a Nanodrop 2000, and 80 ng of DNA was loaded per well for quantitative PCR.

### Fluorescence in situ hybridization (FISH)

Formalin-fixed, paraffin-embedded human and mouse colon tissues were sectioned into 3 µm slices for FISH analysis. *F. nucleatum* was detected using a DNA Bacteria Universal FISH Kit (D-0016, Focobio, Guangzhou, China), following the manufacturer’s instructions. A FITC-labeled oligonucleotide probe specific for *F. nucleatum* (FUS664; 5’-CTT GTA GTT CCG C(C/T) TAC CTC-3’) was applied to tissue sections. After hybridization and washing, fluorescence signals were visualized using a fluorescence microscope (Olympus, Tokyo, Japan). For quantitative assessment, five representative fields per sample were randomly selected at 200 × magnification and evaluated independently by three blinded observers. Based on a previously published cutoff, selected a priori, samples were categorized as low (=20 signals per high power field) or high (>20 signals per high power field) abundance.^25^

### Bacterial strains and growth conditions

*F. nucleatum* (ATCC 25586) was purchased from American Type Culture Collection and cultured under anaerobic conditions (10% H2, 5% CO2, 85% N2) at 37 °C in brain heart infusion (BHI) broth supplemented with hemin, K2HPO4, vitamin K1, and L-cysteine, as previously described.^26^

A fumarate reductase-deficient (*frdA*-KO) strain of *F. nucleatum* was generated following the previously published protocol,^27^ which employs homologous recombination-mediated deletion of the *frdA* gene. Wild-type (WT) and *frdA*-KO strains were cultured under identical anaerobic conditions in BHI culture (BHC). Loss of succinic acid-producing capacity in the *frdA*-KO strain was further confirmed in our laboratory by quantifying succinic acid levels in culture supernatants at matched bacterial densities.

### Mice and induction of colitis

C57BL/6J mice were purchased from Shubeili Biotechnology Co., Ltd. (Wuhan, China) and randomly assigned to experimental groups (n = 5 per group). After a one-week acclimatization period, mice were treated with streptomycin (2 mg/ml) in drinking water for 3 days to reduce endogenous intestinal bacterial load. Subsequently, mice received a daily oral gavage of *F. nucleatum* (10^9^ CFU/ml in phosphate-buffered saline (PBS)) or PBS alone for 2 weeks. Heat-inactivated *F. nucleatum* was prepared by incubation at 95 °C for 25 minutes before gavage.

Experimental colitis was induced by administering 2.5% (w/v) dextran sulfate sodium (DSS) in drinking water ad libitum for 7 days. For the chronic DSS-induced colitis model, mice were administered 2% (w/v) DSS in drinking water for 5 days, followed by autoclaved water for 10 days. This cycle was repeated for three rounds, and mice were euthanized on day 45.^28^ For the TNBS-induced colitis model, mice were presensitized by applying 1.5% TNBS (2508-19-2, Meilunbio, China) in ethanol to the shaved dorsal skin. One week later, mice received intrarectal administration of 100 µL of 2.5% TNBS in ethanol, followed by the corresponding treatments according to the experimental design.^29^

For succinic acid supplementation, 3% (w/v) succinic acid (S9512, Millipore Sigma, USA) was prepared in drinking water and the pH was adjusted to 6.5 with 5 M NaOH. Mice received either 3% succinic acid water or control water starting 3 days before DSS administration.

Disease progression was monitored daily by recording body weight, stool consistency, and fecal occult blood, and then the Disease Activity Index (DAI) was calculated as detailed in Table S6. On day 8 of DSS treatment, mice were euthanized, and colons were removed and measured for length. Tissue samples were collected for various analyzes: a 1 mm segment from the distal colon was processed for transmission electron microscopy; a 5 mm segment was fixed in 4% paraformaldehyde for 24 hours, embedded in paraffin, and sectioned at 3 µm for hematoxylin and eosin (H&E) staining and histological scoring (Table S7). Fresh colon tissues were used for isolation of colonic macrophages and flow cytometric analysis, whereas frozen colon tissues were stored for RNA extraction, Western blotting, and succinic acid quantification.

All experimental procedures were officially approved by the Animal Ethics Committee of Renmin Hospital of Wuhan University (Approval No.: WDRM-20240903A) and conducted in accordance with the institutional guidelines for animal experimentation at Renmin Hospital of Wuhan University.

### Generation of bone marrow-derived macrophages (BMDMs)

BMDMs were generated from 5- to 6-week-old C57BL/6 mice. Femurs and tibias were harvested, and bone marrow was flushed with ice-cold PBS through a 25 G needle. The cell suspension was passed through a 70 µm cell strainer and centrifuged at 250-300 × g for 5 minutes at 4 °C. After discarding the supernatant, red blood cells were lysed with RBC lysis buffer (BL503B, Biosharp, China) according to the manufacturer’s instructions. Cells were then washed twice with PBS and resuspended in RPMI-1640 medium supplemented with 10% fetal bovine serum (FBS) (BC-SE-FBS01, BioChannel, China) and 1% penicillin/streptomycin. Cells were seeded at 1-2 × 10^6^ cells/mL in non-tissue culture-treated bacterial dishes and cultured at 37 °C in a 5% CO2 humidified incubator. M-CSF (RP01216, ABclonal, China) was added on day 0, and the medium was replaced with fresh M-CSF-containing medium on days 3 and 5. After 7 days of differentiation, adherent cells were > 90% CD11b-positive and approximately 80% F4/80-positive, as confirmed by flow cytometry. For *F. nucleatum* treatment, BMDMs were seeded 24 h before stimulation and treated with *F. nucleatum* (MOI = 100:1) for 24 h. For succinic acid treatment, BMDMs were incubated with 0.5 mM succinic acid for 24 h, as previously described.^27^ For NF-κB inhibition, BMDMs were pretreated with BAY 11-7082 (10 µM) for 6 h before stimulation with succinic acid or *F. nucleatum* under LPS-induced inflammatory conditions. Cells were then collected for Western blotting, real-time qPCR, and flow cytometry.

### Enzyme-linked immunosorbent assay (ELISA)

Blood samples were left to clot at room temperature for 2 h and centrifuged at 3000 rpm for 10–20 minutes at 4 °C to obtain serum. Serum aliquots were stored at −80 °C until analysis. Concentrations of tumor necrosis factor-a (TNF-a), interleukin-6 (IL-6), and interleukin-1ß (IL-1ß) were measured using ELISA kits (EM0010, EM0004, EM0029, HUABIO, China) according to the manufacturer’s instructions. All samples were analyzed in triplicate.

### RNA extraction and quantitative real-time PCR (RT-qPCR)

Total RNA was extracted from cells using TRIzol reagent (abs60154, Absin, China) and from mouse colon tissues using the MiniBEST Universal RNA Extraction Kit (9767, TaKaRa, Japan), following the manufacturer’s protocols. RNA concentration and purity were determined with a BioSpectrometer (Eppendorf, Germany). Complementary DNA (cDNA) was synthesized using the Evo M-MLV RT kit (AG11705, Accurate Biology, China). RT-qPCR was performed with Taq Pro Universal SYBR qPCR Master Mix (Q712-02, Vazyme, China) on a CFX Connect Real-Time PCR System (Bio-Rad, USA). ß-actin was used as an internal reference gene, and relative mRNA expression levels were calculated using the 2^−^^ΔΔCt^ method. Primer sequences are listed in Table S8 (Sangon Biotech, China).

### Assessment of intestinal barrier integrity

Mice were fasted for 4 h prior to oral gavage. A freshly prepared solution of fluorescein isothiocyanate-dextran (FITC-dextran, 3-5kDa, 80 mg/mL;60842-46-8, Sigma-Aldrich, USA) in PBS was protected from light until use. Each mouse received 200 µL of FITC-dextran solution by gavage. One hour after administration, abdominal fur was shaved, and fluorescence images were acquired using the IVIS Lumina III system (PerkinElmer, USA) at 470 nm excitation. Plasma samples were collected 4 h after gavage, and fluorescence intensity was measured with a multifunctional plate reader (Ensight, USA) at 485 nm excitation and 530 nm emission.

### Immunofluorescence

For immunofluorescence of cells, coverslips were washed three times with cold PBS, fixed in 4% paraformaldehyde for 20 min at room temperature, permeabilized with 0.5% Triton X-100, and blocked with 5% bovine serum albumin (BSA) for 1 h. For tissue immunofluorescence, paraffin-embedded mouse colon tissue sections (5 µm) were deparaffinized, subjected to antigen retrieval, and blocked with 5% BSA. Sections or cells were incubated with primary antibodies overnight at 4 °C, followed by incubation with secondary antibodies for 1 h at 37 °C in the dark. Nuclei were counterstained with 4’,6-diamidino-2-phenylindole (DAPI; P0131, Beyotime, China), and samples were mounted using an antifluorescence quenching mounting medium (Beyotime, China) before imaging with a fluorescence microscope (Olympus, Japan).

Primary and secondary antibodies used included ZO-1 (ab221547, Abcam, USA), CD3 (ab135372, Abcam, USA), CD4 (ab133616, Abcam, USA), CD8 (ab237709, Abcam, USA), CD68 (ab201340, Abcam, USA), CD83 (ab244204, Abcam, USA), CD86 (ab239075, Abcam, USA), CD206 (ab64693, Abcam, USA), F4/80 (ab90247, Abcam, USA), iNOS (ab3523, Abcam, USA), SUCNR1 (SAB4502428, Sigma-Aldrich, USA), p-p65(82335-1-RR, Proteintech, China), FITC-labeled Goat Anti-Rabbit IgG (H + L) (A0562, Beyotime, China), Cy3-labeled Goat Anti-Rabbit IgG (H + L) (A0516, Beyotime, China).

### Transmission electron microscopy (TEM)

Fresh distal colon tissues (approximately 1 mm^3^) were fixed in 2.5% glutaraldehyde at 4 °C for 24 h, washed three times with 0.1 M phosphate buffer (PB, pH 7.4), and post-fixed in 1% osmium tetroxide for 2 h at room temperature. After three additional PBS washes, samples were dehydrated through a graded ethanol series, infiltrated, and embedded in epoxy resin. Resin blocks were polymerized at 60 °C for 48 h, and ultrathin sections were prepared using an ultramicrotome. Sections were examined and imaged with an HT7800 transmission electron microscope (Hitachi, Japan).

### Alcian blue-periodic acid-schiff (AB-PAS) staining

Paraffin-embedded mouse colon tissues were sectioned at 3 µm thickness and stained using an AB-PAS staining kit (G1285, Solarbio, China) according to the manufacturer’s instructions.

### Immunohistochemistry (IHC)

Paraffin-embedded mouse colon tissues were sectioned at 3 µm thickness for IHC. Staining was performed using the UltraSensitive™ SP (mouse/rabbit) IHC kit (Maxim, China) according to the manufacturer’s instructions. The primary antibody used was anti-MUC2 (ab272692, Abcam, USA).

### Western blotting

Total proteins from tissues and cells were extracted using RIPA lysis buffer (G2002, Servicebio, China) supplemented with 1% phenylmethylsulfonyl fluoride (PMSF; G2008, Servicebio, China), and a phosphatase inhibitor cocktail (G2006, Servicebio, China). Lysates were sonicated and centrifuged at 14,000 rpm for 15 min at 4 °C, and protein concentrations were measured using a BCA assay kit (P0012-1, Beyotime, China). Equal amounts of protein were separated by SDS-PAGE and transferred onto PVDF membranes (E801-01, Vazyme, China). Membranes were blocked with 5% skim milk for 2 h at room temperature, then incubated overnight at 4 °C with primary antibodies diluted in TBST. After washing, membranes were incubated with HRP-linked antibodies (A21010, A21020, Abbkine, China) for 1 h at room temperature. Bands were visualized using the ChemiDoc™ XRS + imaging system (Bio-Rad, USA). Primary antibodies included ZO-1 (21773-1-AP, Proteintech, China), Occludin (27260–1-AP, Proteintech, China), BCL-2 (R23309, ZENBIO, China), Bax (ER0907, HUABIO, China), iNOS (A3774, ABclonal, China), Arg-1 (A4923, ABclonal, China), SUCNR1(31987-1-AP, Proteintech, China), p65 (A19653, ABclonal, China), p-p65 (AP1294, ABclonal, China), IκBa (A19714, ABclonal, China), p-IκBa(AP0707, ABclonal, China), ß-tubulin (80713-1-RR, Proteintech, China), and ß-actin (81119-2-RR, Proteintech, China).

### Flow cytometry

Macrophages from the intestinal lamina propria were isolated as previously described.^30^ Cells were stained according to the respective antibody manufacturers’ protocols. Zombie Aqua viability dye (423102, BioLegend, USA) was used to discriminate live and dead cells. For BMDM, the same staining protocol was applied. Surface markers were detected using anti-CD45 (561141 and 561037, BD Biosciences, USA), anti-F4/80(565409 and 565613, BD Biosciences, USA), anti-CD11b (561082 and 562222, BD Biosciences, USA), anti-CD86(561048 and 560581, BD Biosciences, USA), anti-CD206(561664 and 141707, BD Biosciences and BioLegend, USA), anti-Ly6C (128049, BioLegend, USA), and MHC II(3094413, ThermoFisher, USA). Intracellular markers were detected using anti-TNF-a (506327, BioLegend, USA), anti-iNOS (130-116-357, Miltenyi Biotec, Germany), and anti-Arg1(165803, BioLegend, USA). Flow cytometric acquisition was performed on a Beckman Coulter flow cytometer (Beckman Coulter, USA), and data were analyzed with FlowJo version 10.8.1 (FlowJo LLC, Ashland, OR, USA). Macrophages were defined as the cell population positive for CD45, CD11b, and F4/80 (Figure S1).

### Liquid chromatography-tandem mass spectrometry (LC-MS/MS) analysis of metabolites in *F. nucleatum* culture supernatant

Culture supernatant of *F. nucleatum* (CSF) and corresponding control medium were subjected to untargeted metabolomic profiling by Wuhan Metware Biotechnology Co., Ltd. (Wuhan, China). Samples stored at −80 °C were thawed on ice, and 150 µL of extraction solvent (acetonitrile: methanol = 1:4, v/v) containing internal standards was added to 50 µL of sample. After vortexing for 3 min and centrifugation at 12,000 rpm for 10 min at 4 °C, the supernatant was precipitated at −20 °C for 30 min and centrifuged again. A 120 µL aliquot of the clarified extract was used for LC-MS/MS analysis.

Chromatographic separation was performed on a Waters ACQUITY Premier HSS T3 column (1.8 µm, 2.1 × 100 mm) using a Vanquish UHPLC system (Thermo Fisher). Samples were analyzed in both positive and negative ion modes using a Q Exactive HF-X mass spectrometer (Thermo Fisher Scientific). The mobile phases consisted of 0.1% formic acid in water (A) and acetonitrile (B), with a standard gradient elution. MS data were acquired in data-dependent MS/MS mode over an m/z range of 70–1000.

For untargeted LC-MS/MS metabolomic profiling, four independent *F. nucleatum* culture supernatant samples and three independent BHC samples were analyzed. Each biological replicate was analyzed by a single LC-MS/MS injection. Pooled quality-control (QC) samples were prepared by mixing equal aliquots from all samples and were injected periodically throughout the analytical sequence to monitor instrument stability and data reproducibility.

Raw data were converted to mzXML format using ProteoWizard and processed with an in-house XCMS-based pipeline for peak detection, alignment, and integration. Metabolite annotation was performed using an in-house MS/MS spectral library developed by Wuhan Metware Biotechnology Co., Ltd. The in-house library is proprietary and integrates reference spectra and metabolite information from public databases, including HMDB, KEGG, MoNA, and MassBank.

For two-group comparisons, differential metabolites were identified based on variable importance in projection (VIP > 1), Student’s t-test (*P* < 0.05), and an absolute log2 fold change >1. VIP values were extracted from OPLS-DA models generated with the MetaboAnalystR package after log2 transformation and mean centering of the data. To avoid overfitting, 200-permutation tests were performed for model validation.

### Succinic acid quantification

Succinic acid concentrations in *F. nucleatum* culture supernatants, mouse serum, and colonic tissues were measured using a commercial succinic acid assay kit (S0529S, Beyotime, China) according to the manufacturer’s instructions. Serum was obtained by centrifuging whole blood at 1,000–2,000 × g for 10 min. Colonic tissues were homogenized in BeyoLysis™ Buffer A and centrifuged at 12,000 × g for 3–5 min to obtain supernatant. Culture supernatants were collected directly from bacterial cultures. After addition of the Amplex Red working solution, samples were incubated at 37 °C for 30 min, and absorbance at 570 nm (or fluorescence at Ex/Em = 560/590 nm) was measured. Succinic acid concentrations were calculated from standard curves.

### Cell transfection

Small interfering RNA (siRNA) molecules targeting mouse SUCNR1(siSUCNR1) and a non-targeting control siRNA (siNC) were synthesized by Sangon Biotech (Shanghai, China). BMDMs were transfected with siSUCNR1 using Lipomaster 3000 (TL301-01, Vazyme, China) according to the manufacturer’s protocol. Control groups included cells treated with Lipomaster 3000 alone and cells transfected with siNC. Knockdown efficiency was assessed 48-72 h after transfection by RT-qPCR and Western blotting. siRNA sequences are listed in Table S9.

### Bulk RNA sequencing

BMDMs were randomly divided into three groups: an LPS treatment group, an *F. nucleatum* + LPS treatment group, and an *F. nucleatum* + LPS with siSUCNR1 treatment group (*n* = 3 per group). After the indicated interventions, total RNA was extracted from BMDMs using TRIzol reagent (Invitrogen, USA). The extracted RNA was subsequently subjected to quality assessment, library construction, and high-throughput sequencing. The obtained sequencing data were analyzed using R. Differentially expressed genes (DEGs) between groups were identified using DESeq2, with genes satisfying an adjusted *P* value < 0.05 and an |log2 fold change| > 1.2 defined as DEGs. Based on the experimental design, genes upregulated in the *F. nucleatum* + LPS group relative to the LPS group were intersected with genes downregulated in the *F. nucleatum* + LPS siSUCNR1 group relative to the *F. nucleatum* + LPS group to identify SUCNR1-dependent *F. nucleatum*-responsive genes. The intersecting genes were then subjected to GO enrichment analysis to identify significantly enriched biological processes and signaling pathways.

### Statistical analyzes

Statistical analyzes were performed using GraphPad Prism version 10.0(GraphPad Software, USA). Data are presented as the mean ± SEM from at least three independent experiments. Spearman’s rank correlation was used for correlation analysis. Comparisons between two groups were performed using Student’s t-test or the Mann-Whitney U test, depending on data distribution. For comparisons among three or more groups, one-way ANOVA followed by appropriate post hoc tests was used. A two-sided *P* value less than 0.05 was considered statistically significant. The significance was defined as *P < 0.05, **P < 0.01, ***P < 0.001, and ****P < 0.0001.

## Results

### *F. nucleatum* is abundant in IBD and associated with disease activity and recurrence

To characterize gut microbial alterations in IBD, we first performed high-throughput 16S rRNA gene sequencing of fecal samples from two independent cohorts, including the Renmin cohort and the Renji cohort. Alpha diversity analysis showed that both UC and CD patients exhibited reduced microbial richness and diversity compared with healthy controls (CON), and this trend was consistently observed across the two cohorts (Figure S2).

**Figure 1.**
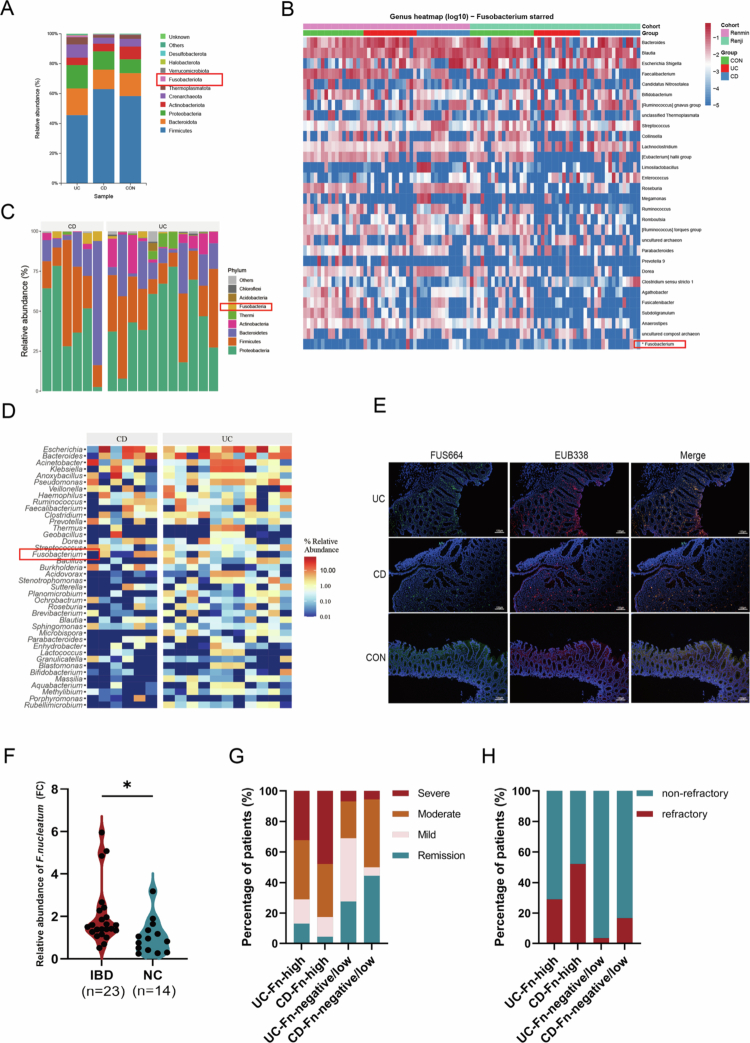
*F. nucleatum* is enriched in patients with IBD and is associated with disease activity. (A) Relative abundance of bacterial communities at the phylum level in fecal samples from healthy controls (CON), UC patients, and CD patients based on high-throughput 16S rRNA gene sequencing. Fecal samples were obtained from two independent cohorts, including the Renmin cohort (CON, *n* = 17; UC, *n* = 15; CD, *n* = 15) and the Renji cohort (CON, *n* = 17; UC, *n* = 12; CD, *n* = 17). Fusobacteriota is highlighted. Taxa representing low abundance were grouped as “Others”. (B) Heatmap showing the relative abundance of bacterial genera in fecal samples from the Renmin and Renji cohorts. Color intensity indicates scaled relative abundance. *Fusobacterium* is highlighted. (C) Relative abundance of bacterial communities at the phylum level in fresh intestinal mucosal biopsy samples from UC (*n* = 11) and CD (*n* = 6) patients. Fusobacteriota is highlighted. (D) Heatmap showing the relative abundance of highly represented genera in mucosal biopsy samples from UC (*n* = 11) and CD (*n* = 6) patients. *Fusobacterium* is highlighted. (E) Representative fluorescence in situ hybridization (FISH) images of FFPE intestinal tissue sections from UC, CD, and normal control (CON) individuals. The FISH cohort included CON (*n* = 10), UC (*n* = 60), and CD (*n* = 41). *F. nucleatum* was detected using the FUS664 probe, and total bacteria were detected using the EUB338 probe. Merged images show probe colocalization. Scale bars, 50 µm. (F) Relative abundance of *F. nucleatum* in fresh colonic mucosal tissues from patients with IBD (*n* = 23) and normal controls (NC, *n* = 14), as determined by real-time PCR. (G) Distribution of disease activity in UC (*n* = 60) and CD (*n* = 41) patients stratified according to *F. nucleatum* abundance based on FISH analysis. (H) Proportion of refractory and non-refractory cases in UC and CD patients stratified according to *F. nucleatum* abundance. Data are presented as mean ± SEM. *P < 0.05, **P < 0.01, ***P < 0.001.

We next examined the relative abundance of *Fusobacteria*-related taxa in fecal samples. At the phylum level, *Fusobacteriota* was significantly enriched in both UC and CD patients compared with CON in the combined cohort analysis (Figure 1A). Consistently, at the genus level, *Fusobacterium* was also significantly increased in UC and CD patients relative to CON (Figure 1B). Stratified analysis by cohort further confirmed that the enrichment of *Fusobacteriota* and *Fusobacterium* was reproducible in both the Renmin and Renji cohorts (Figure S3A-C).

To further determine whether this enrichment was also present in the intestinal mucosal compartment, we performed 16S rRNA sequencing on fresh intestinal mucosal biopsy samples from IBD patients, including UC patients and CD patients. Consistent with the fecal microbiota findings, *Fusobacteriota*, which was relatively low in abundance under non-IBD conditions, was markedly enriched in IBD mucosal tissues and ranked among the dominant phyla (Figure 1C). Similarly, the genus *Fusobacterium* shifted from a minor constituent to a relatively abundant genus in the mucosal microbiota of IBD patients (Figure 1D).

Next, we quantified *F. nucleatum* abundance in mucosal samples from UC, CD and normal control (Tables S10 and S11). As shown in Figure 1E and Figure S3D, *F. nucleatum* levels were significantly higher in UC and CD tissues compared to NC. Real-time PCR validation further confirmed the elevated mucosal burden of *F. nucleatum* in IBD patients relative to NCs (Figure 1F).

Previous studies have shown that *F. nucleatum* levels in the intestine correlate with disease activity and recurrence in IBD^25^ ^,^ ^31^. Notably, patients in the *F. nucleatum*-high group exhibited significantly higher disease activity and recurrence rates compared to the *F. nucleatum*-low group, both in UC and CD cohorts (Figures 1G, H).

### *F. nucleatum* aggravates experimental colitis in mice

**Figure 2.**
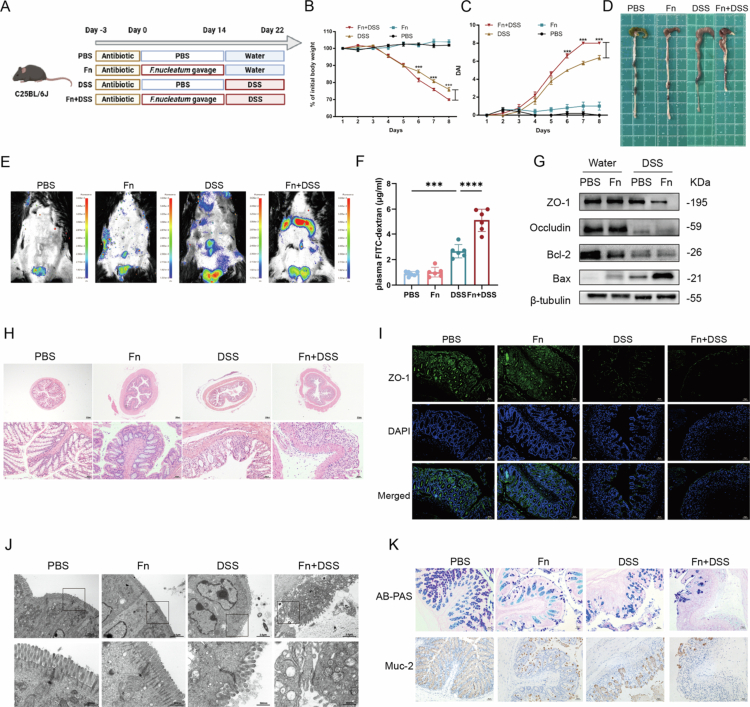
*F. nucleatum* exacerbates DSS-induced colitis and intestinal barrier injury in mice. (A) Experimental workflow. C57BL/6J mice (*n* = 6 per group) were pretreated with streptomycin for 3 days, followed by oral administration of *F. nucleatum* suspended in PBS or PBS alone for 2 weeks. Experimental colitis was then induced by administration of 2.5% DSS in drinking water for 7 days. (B) Percentage change in body weight during DSS-induced colitis. (C) Disease activity index (DAI). (D) Representative images of colon morphology and quantification of colon length. (E) Representative in vivo fluorescence imaging showing FITC-dextran distribution after oral gavage. Red indicates the highest fluorescence intensity and dark purple indicates the lowest intensity. (F) Plasma FITC-dextran concentrations. (G) Western blot analysis of tight junction proteins and apoptosis-related proteins in mouse colon tissues. (H) Representative H&E-stained colon sections. Scale bars: upper panels, 200 µm; lower panels, 50 µm. (I) Representative immunofluorescence staining of ZO-1 (green) and nuclei (DAPI, blue) in colon tissues. Scale bars, 50 µm. (J) Transmission electron microscopy showing ultrastructural alterations of the intestinal epithelial barrier. Scale bars: upper panels, 2 µm; lower panels, 500 nm. (K) Representative AB-PAS staining and MUC2 immunohistochemical staining showing goblet cells and mucin expression. Scale bars: 50 µm. Data are presented as mean ± SEM. ***P < 0.001, ****P < 0.0001.

To further investigate the mechanisms through which *F. nucleatum* contributes to intestinal inflammation, we employed a DSS-induced colitis model that recapitulates key features of mucosal injury (Figure 2A). Fecal 16S rRNA sequencing confirmed that 3-day streptomycin pretreatment markedly altered the endogenous gut microbiota. LEfSe analysis revealed distinct taxonomic profiles before and after streptomycin administration (Figure S4A). Alpha diversity analysis showed a significant reduction in microbial richness after streptomycin treatment, as reflected by the decreased ACE index (Figure S4B). Beta diversity analysis further demonstrated a clear separation of fecal microbial communities before and after streptomycin treatment (Figure S4C). Following this pretreatment, intestinal colonization of *F. nucleatum* was confirmed by fluorescence imaging (Figure S4D).

Compared with DSS treatment alone, mice colonized with *F. nucleatum* exhibited more severe disease, including greater body weight loss (Figure 2B), higher DAI (Figure 2C), and shortened colon length (Figure 2D, Figure S5A). Intestinal barrier permeability assessed using FITC-dextran further showed enhanced extraintestinal fluorescence signals in DSS-treated mice colonized with *F. nucleatum* (Figure 2E). In parallel, plasma FITC-dextran levels were increased 4 h after administration (Figure 2F). Together, these findings indicate aggravated epithelial barrier injury. To further assess epithelial barrier injury and apoptosis in colitis, we examined the expression of tight junction proteins and apoptosis-related markers in colonic tissues. Western blot analysis showed that *F. nucleatum* treatment further reduced the expression of ZO-1 and occludin, decreased the anti-apoptotic protein Bcl-2, and increased the pro-apoptotic protein Bax in colitic mice (Figure 2G and Figure S5B).

Histopathological analysis of colonic tissues revealed that DSS-treated mice colonized with *F. nucleatum* had significantly higher histological scores (Figure S5C), characterized by more severe mucosal damage, crypt destruction, and intense inflammatory cell infiltration (Figure 2H). ZO-1 immunofluorescence showed additional loss of tight junction integrity (Figure 2I), and transmission electron microscopy confirmed more severe ultrastructural disruption of epithelial junctions (Figure 2J). Moreover, goblet cell numbers, mucus secretion, and MUC2 were markedly decreased in *F. nucleatum*-colonized DSS-treated mice (Figure 2K). Consistent with these pathological findings, *F. nucleatum* significantly increased the levels of pro-inflammatory cytokines, including TNF-a, IL-6, and IL-1ß, in both colon tissues and serum (Figure S5D-I). Notably, *F. nucleatum* administration alone did not induce appreciable mucosal inflammation compared with PBS controls, indicating that its pathogenic effects manifest primarily under inflammatory conditions. Collectively, these results demonstrate that *F. nucleatum* exacerbates DSS-induced colitis by amplifying inflammatory responses and compromising both epithelial and mucus barrier integrity, whereas its impact is minimal in the absence of DSS-induced inflammation.

To determine whether this pro-colitic effect was reproducible beyond the acute DSS model, we further assessed the effect of *F. nucleatum* in chronic DSS-induced colitis and TNBS-induced colitis models. In the chronic DSS-induced colitis model, *F. nucleatum* colonization further aggravated colitis severity, as shown by shortened colon length (Figure S6A–B), more severe histological damage (Figure S6C), reduced goblet cell abundance and MUC2 expression (Figure S6D), and weakened and discontinuous ZO-1 fluorescence signals compared with DSS treatment alone (Figure S6E). Similarly, in the TNBS-induced colitis model, *F. nucleatum* administration further exacerbated intestinal inflammation, with greater colon shortening (Figure S6F–G), more pronounced mucosal injury (Figure S6H), reduced goblet cell abundance and MUC2 expression (Figure S6I), and weakened and discontinuous ZO-1 fluorescence signals (Figure S6J) compared with TNBS alone. In contrast, *F. nucleatum* alone did not induce obvious colonic inflammation or barrier injury in either model. Together with the acute DSS-induced colitis findings, these results demonstrate that the pro-colitic effect of *F. nucleatum* is reproducible across multiple colitis models, supporting its broader pathogenic role under inflammatory conditions.

### Mucosal *F. nucleatum* enrichment is associated with macrophage accumulation and a pro-inflammatory macrophage phenotype in IBD tissues

**Figure 3.**
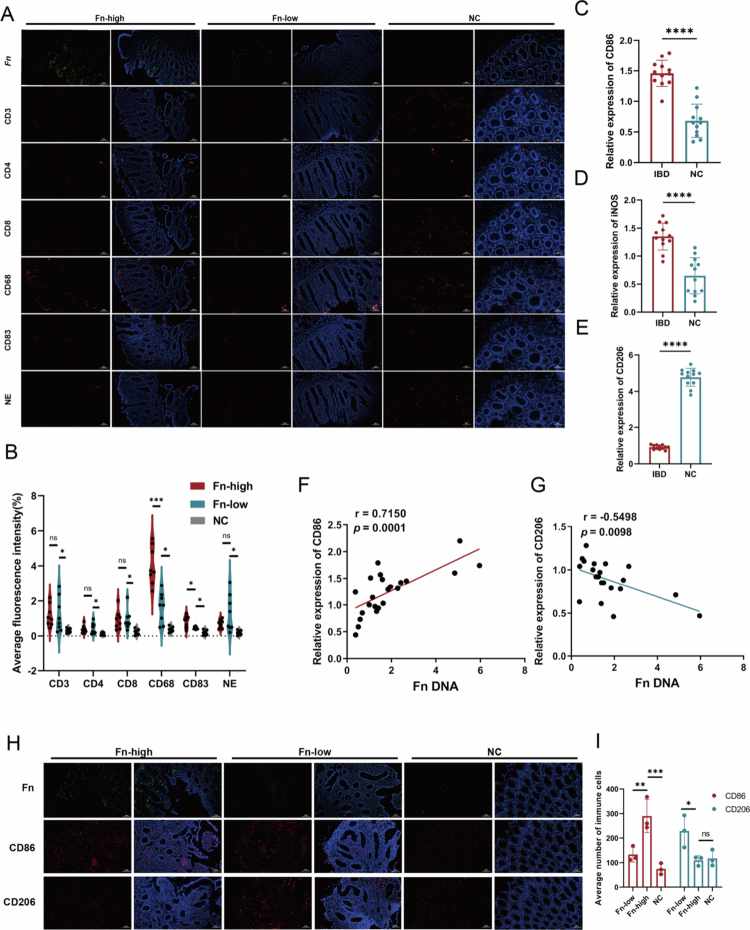
Mucosal *F. nucleatum* enrichment is associated with macrophage accumulation and a pro-inflammatory macrophage phenotype in IBD tissues. (A) Representative immunofluorescence images showing immune cell infiltration in *F. nucleatum*-high, *F. nucleatum*-low IBD tissues, and normal control (NC) tissues using FFPE intestinal tissue sections from the FISH cohort. *F. nucleatum* was detected by FISH. CD3, CD4, and CD8 were used to identify T cell subsets; CD68 was used to identify macrophages; CD83 was used to identify dendritic cells; and NE (neutrophil elastase) was used to identify neutrophils. (B) Quantification of CD3^+^, CD4^+^, CD8^+^, NE^+^, CD68^+^, and CD83^+^ cells in *F. nucleatum*-high IBD tissues, *F. nucleatum*-low IBD tissues, and NC tissues using FFPE sections (*n* = 8 per group). (C–E) RT-qPCR analysis of CD86, NOS2 and CD206 mRNA expression in fresh mucosal biopsies from IBD patients and NC (*n* = 12 per group). (F,G) Spearman correlation between *F. nucleatum* DNA abundance and macrophage-associated markers CD86 and CD206 in fresh mucosal biopsies from IBD patients with paired qPCR data (H) Representative immunofluorescence images showing CD86^+^ and CD206^+^ cells in *F. nucleatum*-high IBD tissues, *F. nucleatum*-low IBD tissues, and NC tissues using FFPE intestinal tissue sections (*n* = 3 per group). (I) Quantification of CD86^+^ and CD206^+^ cells in *F. nucleatum*-high IBD tissues, *F. nucleatum*-low IBD tissues, and NC tissues using FFPE sections. Data are presented as mean ± SEM. Statistical significance: *P < 0.05, **P < 0.01, ***P < 0.001, ****P < 0.0001.

Given that *F. nucleatum* has been reported to modulate immune cell responses during intestinal inflammation in IBD,^18^ ^,^ ^32^ we next investigated its potential association with immune cell infiltration in human colonic tissues. IBD samples were stratified into *F. nucleatum-*high and *F. nucleatum-*low groups based on FISH analysis, and normal control (NC) tissues were included as an additional reference group (Figure 3A). Compared with NC tissues, IBD tissues exhibited increased infiltration of multiple immune cell populations. Among IBD samples, no significant differences were observed in the infiltration of CD3^+^, CD4^+^, or CD8^+^ T cells or neutrophils between the *F. nucleatum*-high and *F. nucleatum*-low groups. In contrast, *F. nucleatum*-high tissues showed markedly higher infiltration of CD68^+^ macrophages and CD83^+^ dendritic cells than *F. nucleatum*-low tissues, with the increase in CD68^+^ macrophages being the most pronounced (Figure 3B). These findings suggest that mucosal *F. nucleatum* enrichment is preferentially associated with increased myeloid cell infiltration, particularly macrophage accumulation, in IBD tissues.

Macrophages exhibit substantial phenotypic heterogeneity in intestinal inflammation, and their functional states are closely associated with colitis severity.^14^ To examine whether *F. nucleatum* affects macrophage-associated inflammatory phenotypes, we quantified the mRNA levels of pro-inflammatory markers(CD86, NOS2) and the repair-associated marker CD206 in colonic tissues from IBD patients and NC by RT-qPCR. The results revealed significant upregulation of CD86 and NOS2 in IBD tissues, whereas CD206 expression was notably downregulated (Figure 3C–E). Furthermore, *F. nucleatum* abundance positively correlated with CD86 expression (Figure 3F) and negatively correlated with CD206 expression (Figure 3G) in IBD tissues.

Immunofluorescence of CD86 and CD206 was further performed in *F. nucleatum*-high IBD tissues, *F. nucleatum*-low IBD tissues, and NC tissues (Figure 3H). Compared with *F. nucleatum*-low tissues and NC tissues, *F. nucleatum*-high IBD tissues showed markedly increased CD86 expression, whereas CD206 expression was relatively reduced. Quantitative analysis further showed that the number of CD86^+^ cells was significantly higher in *F. nucleatum*-high tissues than in *F. nucleatum*-low and NC tissues, while CD206^+^ cells were more abundant in *F. nucleatum*-low tissues than in *F. nucleatum*-high tissues (Figure 3I). These findings suggest that mucosal *F. nucleatum* enrichment is associated with a shift toward a pro-inflammatory macrophage phenotype in IBD tissues.

### *F.* *nucleatum* promotes a pro-inflammatory macrophage phenotype in experimental colitis and BMDMs

**Figure 4.**
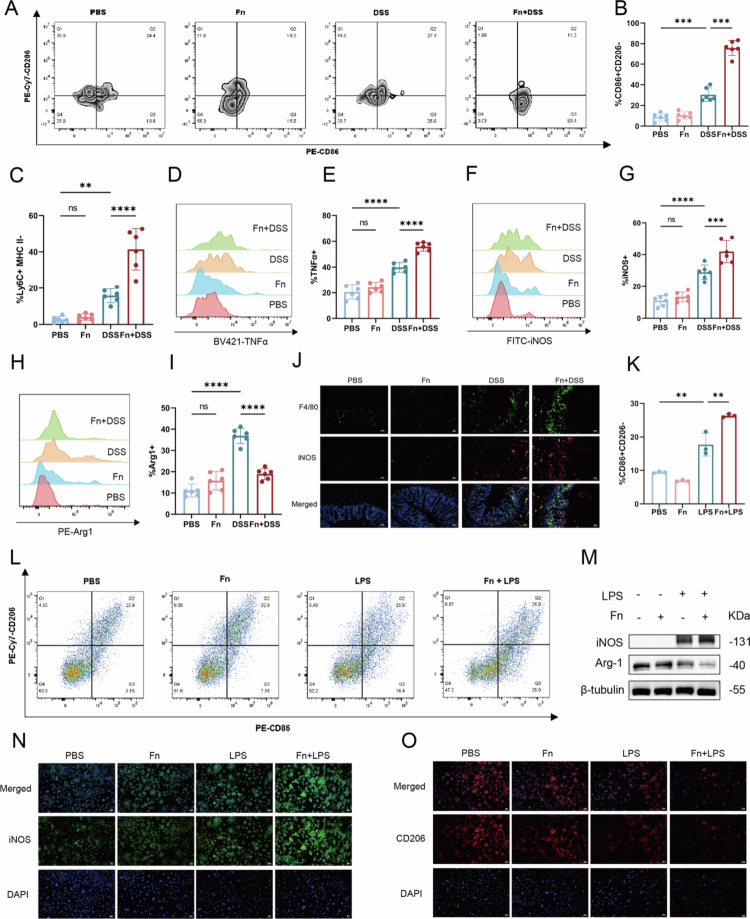
*F. nucleatum* promotes a pro-inflammatory macrophage phenotype during intestinal inflammation. (A) Representative flow cytometry plots showing CD86 and CD206 expression in colonic lamina propria macrophages from PBS-, *F. nucleatum*-, DSS-, and *F. nucleatum* + DSS-treated mice. (B) Quantification of CD86^+^CD206^-^ macrophages in colonic lamina propria.(C) Quantification of Ly6C^+^MHCII^-^ macrophages in colonic lamina propria. (D, E) Representative histogram and quantification of TNF-a expression in intestinal macrophages. (F, G) Representative histogram and quantification of iNOS expression in intestinal macrophages. (H, I) Representative histogram and quantification of Arg1 expression in intestinal macrophages. (J) Representative immunofluorescence staining of F4/80 and iNOS in colonic tissues from the indicated groups.(K) Quantification of CD86^+^CD206^-^ BMDMs under LPS-induced inflammatory conditions. (L) Representative flow cytometry plots showing CD86 and CD206 expression in BMDMs treated with PBS, *F. nucleatum*, LPS, or *F. nucleatum* + LPS.(M) Western blot analysis of iNOS and Arg1 expression in BMDMs from the indicated groups. ß-tubulin was used as the loading control. (N) Representative immunofluorescence staining of iNOS in BMDMs. (O) Representative immunofluorescence staining of CD206 in BMDMs. Data are presented as mean ± SEM. **P < 0.01, ***P < 0.001; ns, not significant.

To further characterize the phenotypic changes of intestinal macrophages induced by *F. nucleatum*, we analyzed maturation-associated markers and inflammatory effector molecules in colonic lamina propria cells. In the acute DSS-induced colitis model, *F. nucleatum* significantly increased the proportion of CD86^+^CD206^−^ macrophages in the colons of DSS-treated mice, suggesting an enhanced pro-inflammatory macrophage phenotype (Figure 4A–B). We then further assessed macrophage heterogeneity by examining Ly6C and MHCII expression within the macrophage population. *F. nucleatum* significantly increased the proportion of Ly6C^+^MHCII^−^ macrophages in DSS-treated mice, indicating the accumulation of an inflammatory macrophage subset with monocyte-like features during DSS-induced colitis (Figure 4C, Figure S7A). Consistently, *F. nucleatum* further increased the expression of the pro-inflammatory mediators TNF-a and iNOS in intestinal macrophages from DSS-treated mice (Figure 4D–G), whereas the repair-associated marker Arg1 was decreased (Figure 4H, I). Immunofluorescence staining of colonic tissues further confirmed increased F4/80^+^iNOS^+^ macrophage accumulation after *F. nucleatum* treatment (Figure 4J).

We next examined whether this macrophage phenotype could be reproduced in other colitis settings. In the chronic colitis model, *F. nucleatum* similarly increased the proportion of CD86^+^CD206^−^ macrophages and enhanced TNF-a and iNOS expression, while reducing Arg1 expression (Figure S7B-I). Consistently, F4/80/iNOS immunofluorescence staining in the TNBS-induced colitis model showed increased iNOS expression in F4/80^+^ macrophages following *F. nucleatum* treatment (Figure S7J). Collectively, these findings indicate that *F. nucleatum* promotes the expansion of inflammatory macrophage subsets and shifts intestinal macrophages toward a pro-inflammatory phenotype while attenuating repair-associated macrophage features during intestinal inflammation.

To further clarify the effect of *F. nucleatum* on macrophage functional phenotypes, we examined BMDMs derived from C57BL/6 mice. In line with our in vivo findings, *F. nucleatum* significantly increased the proportion of CD86^+^CD206^−^ macrophages (Figure 4K–L), enhanced the expression of the pro-inflammatory marker iNOS in BMDMs (Figure 4M–N), and reduced the expression of the repair-associated marker Arg1 and CD206 (Figure 4M, Figure 4O, Figure S7K). Additionally, *F. nucleatum* enhanced the transcription levels of multiple pro-inflammatory mediators, including iNOS, IL-1ß, IL-6, and TNF-a (Figure S7L–P), while reducing the expression of the repair-associated markers CD206 (Figure S7O).

Collectively, these results demonstrate that *F. nucleatum* drives macrophages toward a pro-inflammatory functional state while attenuating repair-associated features under inflammatory conditions, which may contribute to the exacerbation of intestinal inflammation.

#### *F. nucleatum* exacerbates colitis in mice by modulating macrophage functional phenotypes through its small-molecule metabolites

Previous studies have shown that *F. nucleatum* can influence colorectal cancer pathogenesis via the secretion of metabolites.^33^ Building on this, we aimed to investigate whether *F. nucleatum*, through its metabolites produced during colonization, contributes to the onset and progression of colitis. To test this, we administered heat-inactivated *F. nucleatum* to DSS-induced colitis mice. In contrast to live *F. nucleatum*, heat-inactivated *F. nucleatum* did not exacerbate colitis (Figure S8A–F) or promote pro-inflammatory macrophage phenotypes in intestinal macrophages (Figure S8G-H). These findings suggest that *F. nucleatum* primarily exacerbates colonic inflammation through its metabolic products, which drive macrophages toward a pro-inflammatory functional state.

**Figure 5.**
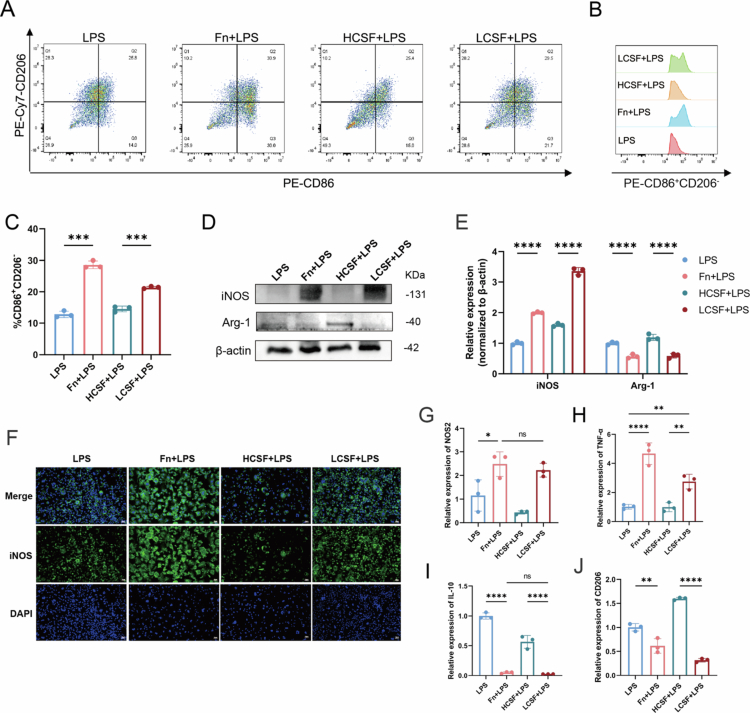
Low-molecular-weight metabolites from *F. nucleatum* culture supernatant promote pro-inflammatory macrophage activation. (A) Representative flow cytometry plots showing CD86 and CD206 expression in BMDMs treated with LPS, *F. nucleatum* + LPS, HCSF + LPS, or LCSF + LPS. (B) Representative histogram showing the CD86^+^CD206^−^ macrophage population in the indicated groups. (C) Quantification of CD86^+^CD206^−^ BMDMs. (D) Western blot analysis of iNOS and Arg-1 expression in BMDMs from the indicated groups. ß-actin was used as the loading control. (E) Quantification of iNOS and Arg-1 protein expression normalized to ß-actin. (F) Representative immunofluorescence staining of iNOS in BMDMs. Nuclei were counterstained with DAPI. (G–H) RT-qPCR analysis of pro-inflammatory mediators, including NOS2 and TNF-a, in BMDMs. (I–J) RT-qPCR analysis of IL-10 and CD206 expression in BMDMs. Data are presented as mean ± SEM. **P < 0.01, ***P < 0.001, ****P < 0.0001; ns, not significant.

To further validate this hypothesis, we fractionated the culture supernatant of *F. nucleatum* (CSF) using a 3 kDa ultrafiltration membrane into low-molecular-weight (LCSF) and high-molecular-weight fractions (HCSF). These fractions were then separately applied to BMDMs to assess their distinct effects. Under equivalent LPS stimulation, BMDMs exposed to LCSF displayed a significantly higher proportion of CD86^+^CD206^−^ macrophages than those treated with HCSF (Figure 5A–C). Consistently, Western blotting, immunofluorescence, and RT-qPCR analyses showed that LCSF treatment markedly enhanced the expression of pro-inflammatory mediators, including iNOS andTNF-a (Figure 5D–H), while suppressing repair-associated markers such as CD206 and IL-10 (Figure 5D–E, I, J). These results indicate that LCSF promotes a pronounced shift toward a pro-inflammatory macrophage phenotype. The overall effects of LCSF treatment were comparable to those observed with direct *F. nucleatum* stimulation.

In summary, our results demonstrate that *F. nucleatum* exacerbates colitis by promoting a shift toward a pro-inflammatory macrophage phenotype, an effect primarily mediated by its small-molecule metabolites.

### Succinic acid is the key metabolite mediating the pro-inflammatory effects of *F. nucleatum*

**Figure 6.**
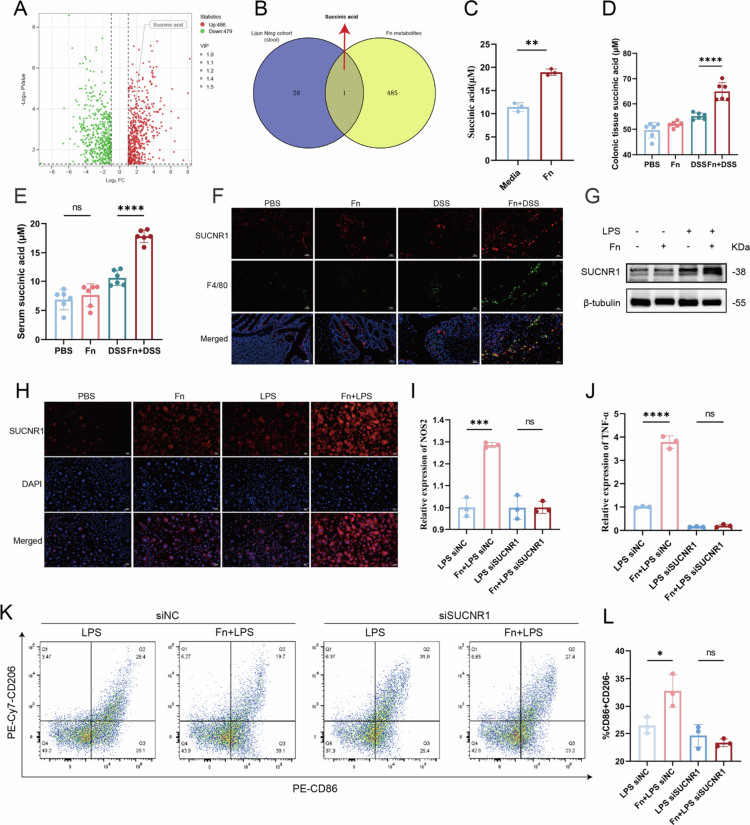
*F. nucleatum*-derived succinic acid promotes pro-inflammatory macrophage activation through SUCNR1. (A) Scatter plot showing differential metabolites between the culture supernatant of *F. nucleatum* (CSF, *n* = 4) and bacterial culture medium control (BHC, *n* = 3). Metabolites enriched in CSF are shown in red, and metabolites decreased relative to BHC are shown in green. (B) Venn diagram showing the overlap between CSF-enriched metabolites and significantly upregulated fecal metabolites in an independent quantitative IBD metabolomics cohort reported by Ning et al. (C) Quantification of succinic acid concentrations in CSF and BHC. (D, E) Succinic acid concentrations in colonic tissues (D) and serum (E) from mice after oral administration of *F. nucleatum* or PBS control (*n* = 6 per group). (F) Representative immunofluorescence images of SUCNR1 (red), F4/80 (green), and nuclei (DAPI, blue) in mouse colon tissues. Scale bars, 50 µm. (G) Western blot analysis of SUCNR1 expression in BMDMs. (H) Representative immunofluorescence images of SUCNR1 (red) and nuclei (DAPI, blue) in BMDMs. Scale bars, 20 µm. (I-J) RT-qPCR analysis of NOS2 and TNF-a mRNA expression in BMDMs. (K) Representative flow cytometry plots showing CD86 and CD206 expression in BMDMs. (L) Quantification of CD86^+^CD206^−^ BMDMs. Data are presented as mean ± SEM. *P < 0.05, **P < 0.01, ***P < 0.001, ****P < 0.0001.

To identify the small-molecule metabolites released by *F. nucleatum* that promote pro-inflammatory macrophage activation and exacerbate colitis, we performed untargeted LC-MS/MS analysis of the CSF. This analysis identified 486 metabolites that were significantly enriched in the CSF compared to the BHC (Figure 6A). To evaluate their clinical relevance, we compared these CSF-enriched metabolites with significantly upregulated fecal metabolites from an independent quantitative IBD metabolomics cohort reported by Ning et al.^34^ This comparison identified succinic acid as the shared metabolite between *F. nucleatum*-derived metabolites and IBD-enriched fecal metabolites (Figure 6B), suggesting that succinic acid may be a clinically relevant mediator linking *F. nucleatum* colonization to intestinal inflammation.

We then quantified succinic acid levels using a targeted assay, confirming that succinic acid was present at significantly higher concentrations in CSF compared to BHC (Figure 6C). To assess whether these elevated succinic acid levels observed in vitro were also present in vivo, we quantified succinic acid in intestinal tissues and serum from mice following oral administration of *F. nucleatum*. Consistent with our metabolomic data, *F. nucleatum* treatment markedly increased succinic acid concentrations in both the intestine and systemic circulation under inflammatory conditions (Figure 6D, E).

Succinic acid signals through its cognate receptor SUCNR1.^35^ Given the elevated succinic acid levels induced by *F. nucleatum,* we examined SUCNR1 expression in intestinal macrophages. Double immunofluorescence for F4/80 and SUCNR1 revealed significantly increased SUCNR1 expression on intestinal macrophages in *F. nucleatum*-treated mice under inflammatory conditions (Figure 6F). Similarly, BMDMs exposed to *F. nucleatum* exhibited upregulation of SUCNR1 (Figure 6G–H, Figure S9A).

To determine whether SUCNR1 contributes to the pro-inflammatory activation of macrophages induced by *F. nucleatum* under inflammatory conditions, we transfected BMDMs with siRNA targeting SUCNR1 (siSUCNR1) and assessed their response to *F. nucleatum* stimulation (Figure S9B, C). RT-qPCR (Figure 6I–J) and flow cytometry (Figure 6K–L) showed that SUCNR1 knockdown substantially attenuated the *F. nucleatum*-induced pro-inflammatory macrophage activation under inflammatory conditions.

In summary, these findings identify succinic acid as the key metabolite through which *F. nucleatum* promotes a pro-inflammatory macrophage phenotype in the intestine, in part through activation of its cognate receptor, SUCNR1.

### Succinic acid promotes a pro-inflammatory macrophage phenotype to exacerbate colitis

**Figure 7.**
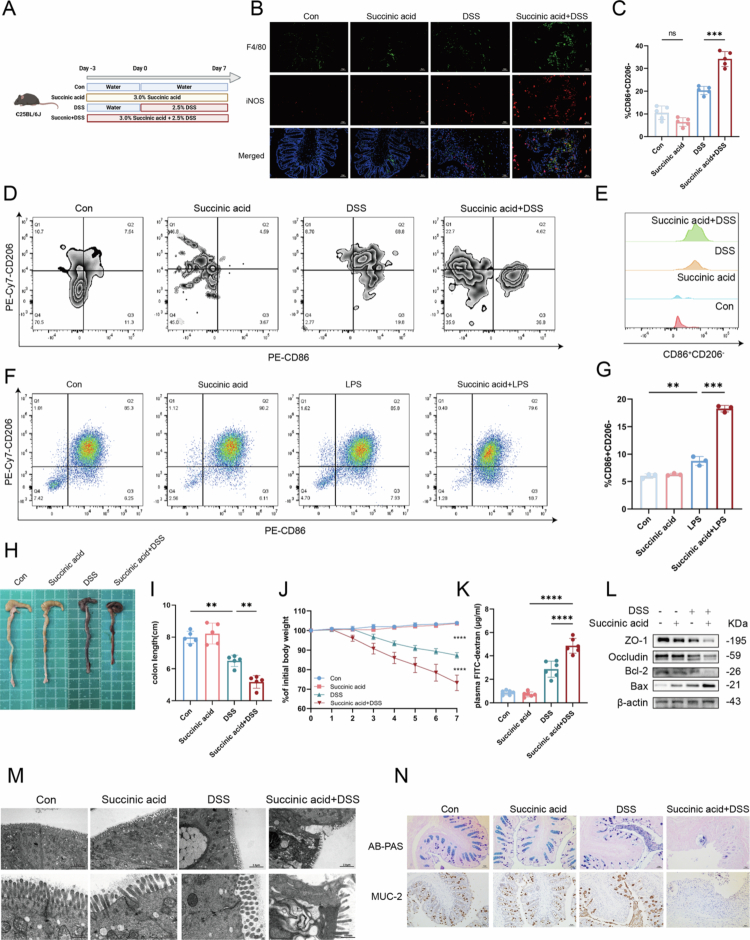
Succinic acid exacerbates DSS-induced colitis by promoting a pro-inflammatory macrophage phenotype and impairing intestinal barrier integrity. (A) Experimental design. C57BL/6J mice (*n* = 5 per group) were provided with 3% (w/v) succinic acid-supplemented drinking water or control water starting 3 days before DSS exposure. Colitis was induced by administering 2.5% (w/v) DSS in drinking water for 7 days, with or without concurrent succinic acid supplementation. (B) Representative immunofluorescence staining of F4/80 (green), iNOS (red), and nuclei (DAPI, blue) in colon tissues. Scale bars, 50 µm. (C, D) Representative flow cytometry plots and quantification of CD86^+^CD206^−^ macrophages in colonic lamina propria from the indicated groups. (E) Representative histogram showing CD86^+^CD206^−^ macrophages in mouse intestinal tissues under different treatments. (F, G) Representative flow cytometry plots and quantification of CD86^+^CD206^−^ BMDMs under LPS-induced inflammatory conditions. (H, I) Representative images of colon morphology and quantification of colon length. (J) Percentage change in body weight during DSS-induced colitis. (K) Plasma FITC-dextran concentrations. (L) Western blot analysis of tight junction proteins and apoptosis-related proteins in mouse colon tissues. (M) Transmission electron microscopy images showing ultrastructural alterations in the intestinal epithelial barrier. Scale bars: upper panels, 2 µm; lower panels, 500 nm. (N) Representative AB-PAS staining and MUC2 immunohistochemical staining showing goblet cells and mucin expression. Scale bars, 50 µm. Data are presented as mean ± SEM. **P < 0.01, ***P < 0.001, ****P < 0.0001.

Given that succinic acid emerged as the key metabolite mediating *F. nucleatum*-induced macrophage reprogramming, we next investigated whether exogenous succinic acid could independently promote a pro-inflammatory macrophage phenotype and exacerbate DSS-induced colitis in C57BL/6J mice (Figure 7A). As expected, immunofluorescence revealed that exogenous succinic acid supplementation significantly increased the abundance of iNOS^+^ intestinal macrophages in DSS-treated mice (Figure 7B). Flow cytometric analysis confirmed that succinic acid notably increased the proportion of CD86^+^CD206^−^ macrophages in the intestinal lamina propria of DSS-treated mice (Figure 7C–E). In vitro assays also showed that succinic acid markedly elevated the proportion of CD86^+^CD206^−^ BMDMs under LPS-induced inflammatory conditions (Figure 7F–G). Furthermore, succinic acid upregulated pro-inflammatory mediators, including NOS2, IL-6, and TNF-a (Figure S10A-C, E, F), while reducing markers commonly associated with anti-inflammatory macrophage phenotype, including CD206 and Arg-1 (Figure S10D, F–G). These results collectively demonstrate that succinic acid is sufficient to skew macrophages toward a pro-inflammatory phenotype under inflammatory conditions.

Consistent with its role in promoting a pro-inflammatory macrophage phenotype, succinic acid further aggravated DSS-induced colonic injury, as reflected by significantly shortened colon length (Figure 7H–I), increased body weight loss (Figure 7J), and elevated DAI scores (Figure S10H) compared with DSS treatment alone. Histological examination confirmed that succinic acid supplementation exacerbated DSS-induced colonic damage, characterized by heightened inflammatory cell infiltration, pronounced crypt loss, and extensive mucosal ulceration (Figure S10I). Additionally, FITC-dextran permeability tests indicated that succinic acid supplementation increased plasma FITC-dextran levels in DSS-treated mice, demonstrating exacerbated epithelial permeability (Figure 7K). Western blot analysis revealed that succinic acid, in combination with DSS, significantly reduced the expression of tight junction proteins ZO-1 and occludin, as well as the anti-apoptotic protein Bcl-2, while increasing the pro-apoptotic protein Bax levels in colonic tissues (Figure 7L, Figure S10J). Transmission electron microscopy further confirmed that succinic acid aggravated ultrastructural damage to the intestinal epithelial barrier in DSS-treated mice (Figure 7M).

Moreover, succinic acid supplementation suppressed goblet cell mucin expression, as observed by AB-PAS staining and MUC2 immunohistochemistry (Figure 7N), and reduced ZO-1 expression in the colon (Figure S10K). Additionally, succinic acid amplified systemic inflammation in DSS-treated mice, reflected by elevated serum levels of IL-6, TNF-a, and IL-1ß (Figure S10L-N).

Taken together, these findings demonstrate that succinic acid promotes a pro-inflammatory activation state of intestinal macrophages under inflammatory conditions, thereby exacerbating the severity of DSS-induced colitis.

### Loss of succinic acid production abolishes the ability of *F. nucleatum* to induce pro-inflammatory macrophage activation and exacerbate colitis

**Figure 8.**
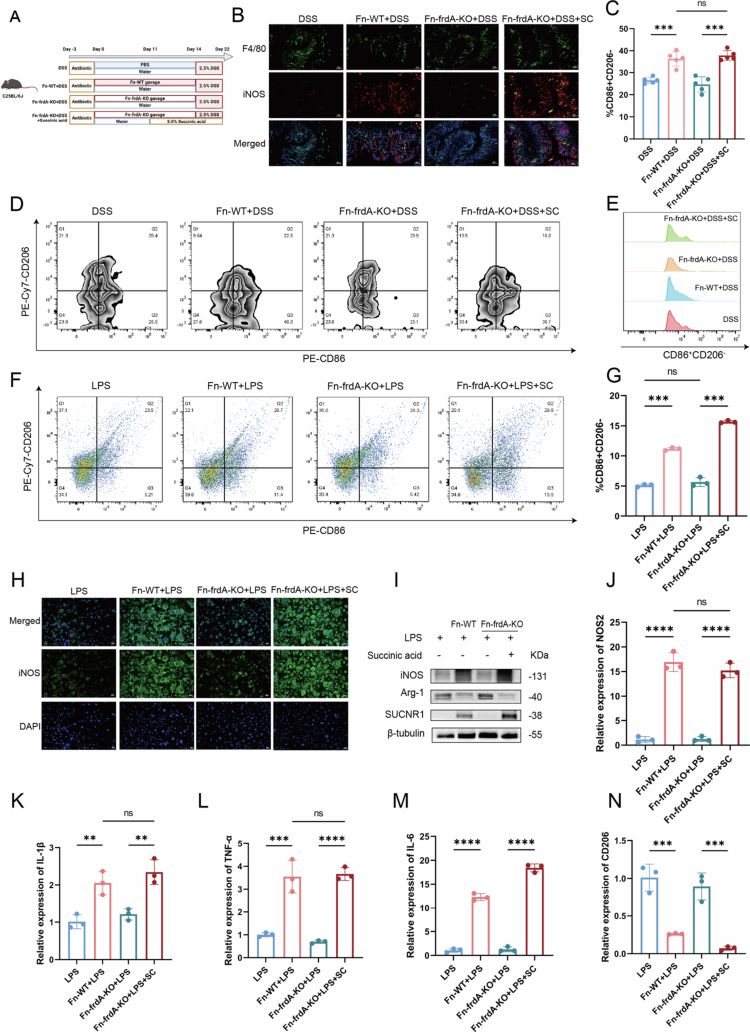
Succinic acid restores the impaired pro-inflammatory macrophage activation induced by the *frdA*-KO *F. nucleatum* strain. (A) Experimental design. C57BL/6J mice (*n* = 5 per group) were pretreated with antibiotics for 3 days, followed by oral gavage of wild-type *F. nucleatum* (Fn-WT), *frdA*-KO *F. nucleatum*, or PBS beginning on Day 0. Colitis was induced by administering 2.5% (w/v) DSS in drinking water from Day 14 to Day 22. Succinic acid supplementation (3%, w/v) was initiated 3 days before DSS exposure and continued throughout DSS treatment. (B) Representative immunofluorescence staining of F4/80 (green), iNOS (red), and nuclei (DAPI, blue) in colon tissues. (C, D) Representative flow cytometry plots and quantification of CD86^+^CD206^−^ macrophages in colonic lamina propria. (E) Representative histogram showing CD86^+^CD206^−^ macrophages in mouse intestinal tissues under different treatments. (F, G) Representative flow cytometry plots and quantification of CD86^+^CD206^−^ BMDMs treated with LPS, Fn-WT + LPS, *frdA*-KO *F. nucleatum* + LPS, or *frdA*-KO *F. nucleatum* + succinic acid + LPS. (H) Representative immunofluorescence staining of iNOS (green) and nuclei (DAPI, blue) in BMDMs. (I) Western blot analysis of iNOS, Arg-1, and SUCNR1 expression in BMDMs. ß-tubulin was used as the loading control. (J–N) RT-qPCR analysis of NOS2, IL-1ß, TNF-a, IL-6, and CD206 mRNA expression in BMDMs. Data are presented as mean ± SEM. **P < 0.01, ***P < 0.001, ****P < 0.0001; ns, not significant.

*F. nucleatum* primarily synthesizes succinic acid via the reductive branch of the tricarboxylic acid (TCA) cycle, catalyzed by fumarate reductase (*frd*) under anaerobic conditions.^36^ To assess the role of succinic acid in *F. nucleatum*-mediated exacerbation of intestinal inflammation, we utilized an *frdA*-deficient strain (*frdA*-KO) of *F. nucleatum*, which lacks succinic acid production. Mice were gavaged with either WT or *frdA*-KO *F. nucleatum* before 2.5% DSS administration. An additional group received *frdA*-KO *F. nucleatum* with exogenous succinic acid supplementation (Figure 8A).

Immunofluorescence revealed that gavage with the *frdA*-KO *F. nucleatum* led to a significantly reduced number of iNOS^+^ macrophages in the intestinal mucosa compared to WT *F. nucleatum*. Notably, succinic acid supplementation restored iNOS^+^ macrophage accumulation to levels comparable to those induced by the WT *F. nucleatum* strain (Figure 8B), confirming that succinic acid mediates the pro-inflammatory macrophage activation. Flow cytometry analysis corroborated these findings, showing a significantly lower proportion of CD86^+^CD206^−^ macrophages in the intestines of *frdA*-KO *F. nucleatum*-treated mice compared to WT *F. nucleatum* treatment. Supplementation with succinic acid restored the frequency of CD86^+^CD206^−^ macrophages to WT levels (Figure 8C–E). In vitro experiments mirrored these results, showing reduced pro-inflammatory macrophage activation in *frdA*-KO *F. nucleatum*-treated BMDMs, which was restored by succinic acid supplementation (Figure 8F–H).

Furthermore, Western blot and RT-qPCR analyses confirmed that *frdA*-KO *F. nucleatum* did not induce a pro-inflammatory macrophage activation, as evidenced by the absence of upregulation of pro-inflammatory markers (iNOS, IL-1ß, TNF-a, IL-6) and the failure to suppress anti-inflammatory markers (Arg-1, CD206) in BMDMs. Supplementation with succinic acid restored the expression pattern of these markers to levels comparable to WT *F. nucleatum* treatment (Figure 8I–N, Figure S11A). Additionally, Western blot analysis showed that the *frdA*-KO strain failed to increase SUCNR1 expression on BMDMs, while succinic acid supplementation restored SUCNR1 levels to those seen with the WT strain (Figure 8I, Figure S11A). Collectively, these results indicate that *F. nucleatum* drives pro-inflammatory macrophage activation in a succinic acid-dependent manner.

**Figure 9.**
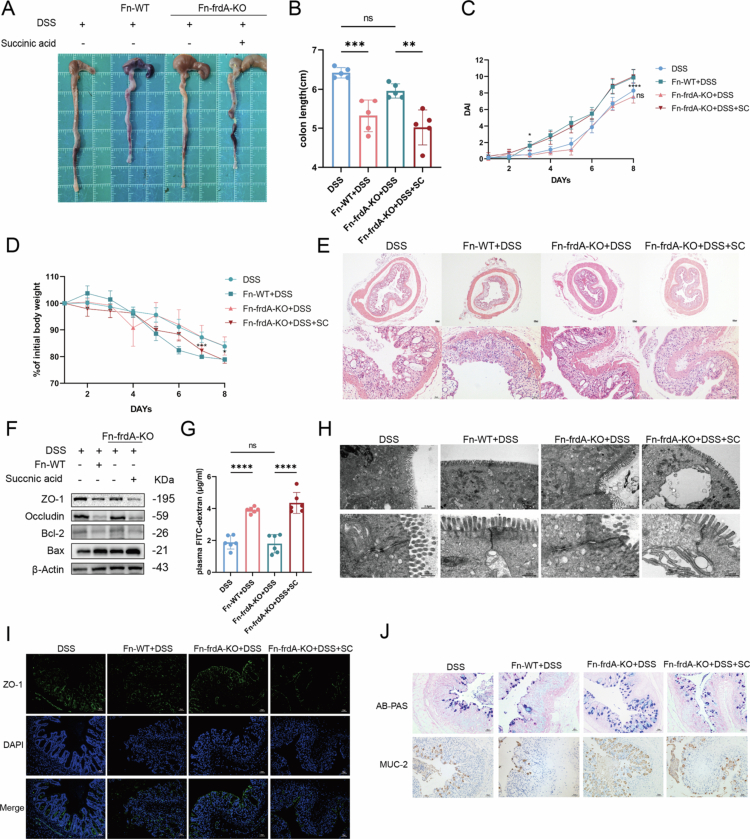
Succinic acid supplementation restores the ability of *frdA*-KO *F. nucleatum* to exacerbate DSS-induced colitis and intestinal barrier injury. (A) Representative images of colon morphology from mice treated with DSS, Fn-WT + DSS, *frdA*-KO *F. nucleatum* + DSS, and *frdA*-KO *F. nucleatum* + DSS + succinic acid. (B) Quantification of colon length. (C) Disease activity index (DAI) during DSS-induced colitis. (D) Percentage change in body weight. (E) Representative H&E-stained colon sections showing histopathological changes. (F) Western blot analysis of tight junction proteins ZO-1 and occludin, the anti-apoptotic protein Bcl-2, and the pro-apoptotic protein Bax in colonic tissues. ß-actin was used as the loading control. (G) Plasma FITC-dextran concentrations. (H) Transmission electron microscopy showing ultrastructural alterations of the intestinal epithelial barrier. Scale bars: upper panels, 2 µm; lower panels, 500 nm. (I) Representative immunofluorescence staining of ZO-1 in colonic tissues. Nuclei were counterstained with DAPI. (J) Representative AB-PAS staining and MUC2 immunohistochemical staining showing goblet cells and mucin expression. Data are presented as mean ± SEM. **P < 0.01, ***P < 0.001, ****P < 0.0001; ns, not significant.

Consistent with its impaired ability to induce pro-inflammatory macrophage activation, gavage with *frdA*-KO *F. nucleatum* did not exacerbate DSS-induced colitis. Mice colonized with *frdA*-KO *F. nucleatum* exhibited colon length, body weight loss, DAI, and histopathological features comparable to the DSS-only group (Figure 9A–E). Western blot analysis further showed that *frdA*-KO *F. nucleatum* failed to affect the expression of tight junction proteins (ZO-1, occludin) or apoptosis-related regulators (Bcl-2, Bax) during DSS treatment (Figure 9F, Figure S11B).

Assessments of epithelial barrier function using plasma FITC-dextran permeability (Figure 9G), transmission electron microscopy (Figure 9H), and ZO-1 immunofluorescence (Figure 9I) showed no significant difference between mice treated with *frdA*-KO *F. nucleatum* and DSS-only controls, indicating that the *frdA*-KO strain failed to aggravate DSS-induced epithelial barrier injury. Similarly, AB-PAS staining and MUC2 immunohistochemistry revealed that *frdA*-KO *F. nucleatum* did not suppress goblet cell numbers or mucin production (Figure 9J). Serum ELISA also revealed no significant increase in systemic inflammatory cytokines following *frdA*-KO *F. nucleatum* gavage (Figure S11C-E).

Importantly, when mice colonized with frdA-KO *F. nucleatum* were given 3.0% succinic acid in drinking water, both intestinal and systemic inflammation were restored to, or exceeding, those observed in WT *F. nucleatum*-treated mice (Figure 9).

In summary, these findings demonstrate that the inability of *F. nucleatum* to produce succinic acid impairs its ability to induce pro-inflammatory macrophage activation and exacerbate colitis. Replenishing succinic acid restores these effects, underscoring the critical role of succinic acid in *F. nucleatum-*induced macrophage reprogramming and intestinal inflammation.

### *F. nucleatum*-derived succinic acid activates SUCNR1-dependent NF-κB signaling in macrophages

**Figure 10.**
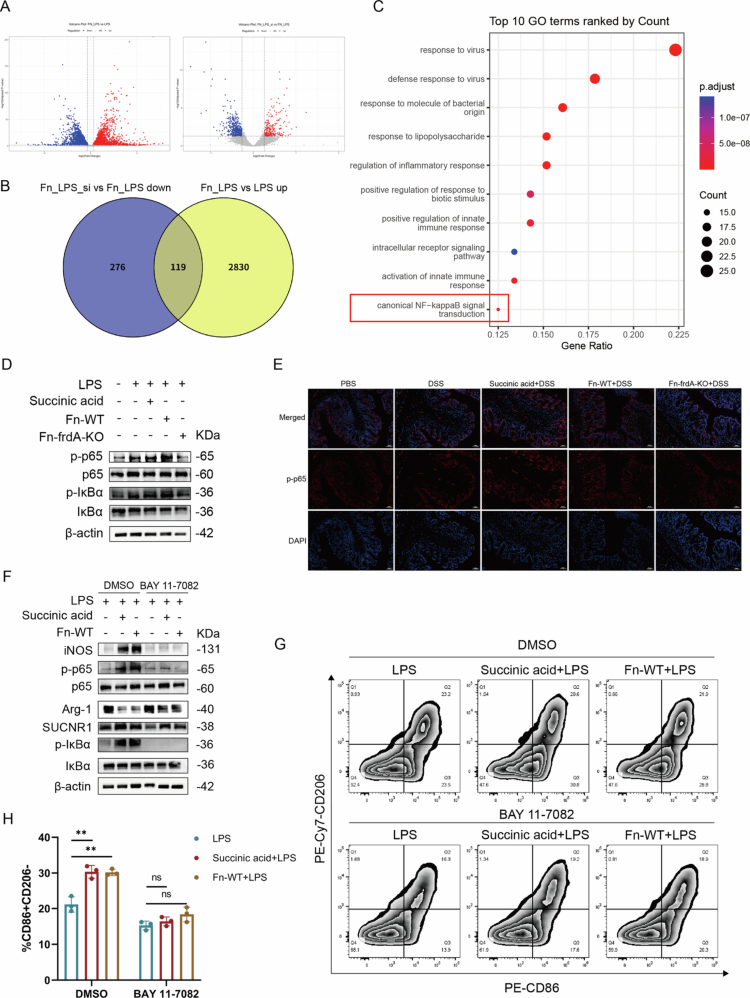
*F. nucleatum*-derived succinic acid activates SUCNR1-dependent NF-κB signaling to promote pro-inflammatory macrophage activation. (A) Volcano plots showing differentially expressed genes in BMDMs treated with *F. nucleatum* + LPS versus LPS and *F. nucleatum* + LPS siSUCNR1 versus *F. nucleatum* + LPS. (B) Venn diagram showing the intersection between genes upregulated by *F. nucleatum* + LPS relative to LPS and genes downregulated after SUCNR1 knockdown in the *F. nucleatum* + LPS background. (C) GO enrichment analysis of the 119 overlapping genes. The top 10 GO terms ranked by gene count are shown. Canonical NF-κB signal transduction is highlighted. (D) Western blot analysis of p-p65, total p65, p-IκBa, and total IκBa in BMDMs treated with LPS, succinic acid + LPS, wild-type *F. nucleatum* + LPS, or *frdA*-KO *F. nucleatum* + LPS. ß-actin was used as the loading control. (E) Representative immunofluorescence staining of p-p65 in colonic tissues from PBS-, DSS-, succinic acid + DSS-, wild-type *F. nucleatum* + DSS-, and *frdA*-KO *F. nucleatum* + DSS-treated mice. Nuclei were counterstained with DAPI. (F) Western blot analysis of iNOS, p-p65, total p65, Arg-1, SUCNR1, p-IκBa, and total IκBa in BMDMs treated with LPS, succinic acid + LPS, or wild-type *F. nucleatum* + LPS in the presence of DMSO or the NF-κB inhibitor BAY 11-7082. ß-actin was used as the loading control. (G) Representative flow cytometry plots showing CD86 and CD206 expression in BMDMs treated with LPS, succinic acid + LPS, or wild-type *F. nucleatum* + LPS in the presence of DMSO or BAY 11-7082. (H) Quantification of the proportion of CD86^+^CD206^−^ BMDMs in the indicated groups. Data are presented as mean ± SEM. **P < 0.01; ns, not significant.

To identify SUCNR1-dependent transcriptional programs induced by *F. nucleatum* under inflammatory conditions, we performed RNA sequencing of BMDMs treated with LPS, *F. nucleatum* + LPS, or *F. nucleatum* + LPS following SUCNR1 knockdown. Differential expression analysis revealed substantial transcriptomic changes between the *F. nucleatum* + LPS group and the LPS group, as well as between the *F. nucleatum* + LPS siSUCNR1 group and the *F. nucleatum* + LPS group (Figure 10A). We then intersected genes upregulated by *F. nucleatum* + LPS relative to LPS with genes downregulated after SUCNR1 knockdown in the *F. nucleatum* + LPS background. This analysis identified 119 overlapping genes, representing a subset of *F. nucleatum*-induced inflammatory genes that were reversed by SUCNR1 knockdown (Figure 10B). GO enrichment analysis showed that these overlapping genes were significantly enriched in immune- and inflammation-related biological processes, including response to virus, response to lipopolysaccharide, regulation of inflammatory response, and positive regulation of innate immune response. Notably, canonical NF-κB signal transduction was also enriched among these genes (Figure 10C), suggesting that SUCNR1 contributes to *F. nucleatum*-induced pro-inflammatory macrophage activation at least partly through NF-κB signaling.

We next examined whether the SUCNR1-dependent transcriptional changes were associated with activation of the NF-κB signaling pathway. In BMDMs under LPS-induced inflammatory conditions, both succinic acid and WT *F. nucleatum* markedly increased the phosphorylation of p65 and IκBa, whereas total p65 and IκBa levels remained relatively unchanged. In contrast, the *frdA*-KO strain, which lacks succinic acid production, failed to induce comparable phosphorylation of p65 or IκBa (Figure 10D, Figure S11F). Consistently, immunofluorescence staining of colonic tissues from DSS-treated mice showed enhanced p-p65 signals following succinic acid supplementation or WT *F. nucleatum* colonization, whereas this activation was markedly attenuated in mice colonized with the *frdA*-KO strain (Figure 10E). These findings indicate that *F. nucleatum*-derived succinic acid activates NF-κB signaling under inflammatory conditions.

To further determine whether NF-κB signaling is required for succinic acid- or *F. nucleatum*-induced macrophage activation, BMDMs were treated with the NF-κB inhibitor BAY 11-7082 under LPS-induced inflammatory conditions. Western blot analysis showed that BAY 11-7082 markedly suppressed p65 and IκBa phosphorylation without substantially altering SUCNR1 expression. NF-κB inhibition attenuated the induction of the pro-inflammatory marker iNOS and restored the expression of Arg-1, suggesting that NF-κB activation is required for succinic acid- and WT *F. nucleatum*-induced pro-inflammatory macrophage reprogramming (Figure 10F, Figure S11G).

Consistently, flow cytometric analysis showed that both succinic acid and WT *F. nucleatum* increased the proportion of CD86^+^CD206^−^ BMDMs under DMSO-treated conditions. However, BAY 11-7082 treatment largely reversed this increase, and the proportions of CD86^+^CD206^−^ cells were no longer significantly different among the LPS, succinic acid + LPS, and WT *F. nucleatum* + LPS groups (Figure 10G–H). Together, these results indicate that NF-κB signaling contributes to succinic acid–SUCNR1-dependent pro-inflammatory macrophage activation.

## Discussion

This study identifies *F. nucleatum* enrichment as a microbiota-associated feature of IBD and demonstrates that *F. nucleatum* aggravates intestinal inflammation through a succinic acid–SUCNR1–NF-κB–macrophage axis. In both fecal samples and intestinal tissues from patients with IBD, *F. nucleatum* and Fusobacterium-related taxa were enriched and were associated with disease activity, refractory disease, and macrophage-associated inflammatory features. In acute DSS-induced, chronic DSS-induced, and TNBS-induced experimental colitis models, *F. nucleatum* consistently exacerbated colitis severity and promoted pro-inflammatory macrophage activation. Mechanistically, *F. nucleatum*-derived succinic acid induced SUCNR1 expression, activated NF-κB signaling, and shifted macrophages toward a pro-inflammatory phenotype. Loss of succinic acid production in the *frdA*-KO strain markedly attenuated macrophage activation and colitis aggravation, whereas exogenous succinic acid restored these effects. Together, these findings support succinic acid as a functionally important microbial metabolite linking *F. nucleatum* colonization to SUCNR1-dependent NF-κB activation and macrophage-mediated intestinal inflammation.

A substantial body of work has implicated succinic acid in intestinal inflammation and IBD.^37^ Succinic acid is not only a tricarboxylic acid cycle intermediate but also an extracellular signaling metabolite that acts through SUCNR1/GPR91.^38^ Previous studies have reported increased succinate levels and SUCNR1 expression in Crohn’s disease tissues and have shown that SUCNR1 contributes to experimental intestinal inflammation and fibrosis.^39^ In immune cells, succinic acid has also been linked to inflammatory metabolic reprogramming, HIF-1a stabilization, IL-1ß production, and maintenance of pro-inflammatory macrophage states^39-41^. Thus, the broad concept that succinic acid can promote inflammatory responses in colitis is already established. However, the microbial source of pathogenic succinic acid, the specific bacterial taxa responsible, and the downstream immune-cell program through which microbiota-derived succinic acid aggravates IBD remain incompletely defined. Our study addresses this gap by identifying *F. nucleatum*-derived succinic acid as a bacterial metabolite that directly engages SUCNR1-dependent NF-κB signaling in macrophages to amplify intestinal inflammation.

The role of *F. nucleatum* in intestinal disease has also been extensively investigated, particularly in colorectal cancer and, more recently, in IBD. Previous studies have shown that *F. nucleatum* can adhere to intestinal epithelial cells, disrupt epithelial barrier integrity, induce mucosal inflammatory responses, and reshape the local immune microenvironment.^42^ Recent evidence further suggests that metabolic reprogramming is an important mechanism underlying *F. nucleatum*–host interactions. For example, one study reported that *F. nucleatum* exacerbates colitis by promoting acetyl-CoA accumulation and activating epithelial STAT3 signaling.^25^ Another study showed that *F. nucleatum*-derived succinic acid contributes to tumor immune resistance in colorectal cancer, establishing succinate as a functionally relevant metabolite produced by this organism.^27^

Our study extends this body of work by shifting the focus from epithelial signaling to macrophage-mediated inflammation in IBD. We show that succinic acid derived from *F. nucleatum* promotes pro-inflammatory macrophage activation through the SUCNR1–NF-κB pathway, thereby aggravating experimental colitis. These findings identify a distinct *F. nucleatum*–succinic acid–SUCNR1–NF-κB axis and provide additional mechanistic insight into how microbial metabolites contribute to intestinal inflammation.

A key advance of this study is the integration of bacterial genetic manipulation, metabolite rescue, and host signaling validation. The *frdA*-KO strain, which exhibited impaired succinic acid production as previously reported, showed a markedly reduced capacity to induce macrophage inflammatory responses and exacerbate experimental colitis. Importantly, exogenous succinic acid restored both macrophage activation and colitis aggravation in the *frdA*-KO setting. These findings support a causal role for bacterial succinate production in promoting intestinal inflammation. In addition, RNA sequencing, pathway enrichment, Western blotting, immunofluorescence, and pharmacological inhibition collectively identified NF-κB signaling as a downstream pathway involved in succinic acid–SUCNR1-dependent macrophage inflammatory activation. This provides a more defined intracellular signaling cascade than previous studies that primarily described succinate accumulation or SUCNR1 involvement in colitis at the tissue level.

Our findings also link *F. nucleatum*–derived succinic acid and macrophage inflammatory responses to epithelial barrier injury, a central pathological feature of active colitis. In our experimental models, *F. nucleatum* or exogenous succinic acid promoted pro-inflammatory macrophage activation, reduced the expression of tight junction proteins, decreased goblet cell abundance and MUC2 expression, increased epithelial apoptosis, and enhanced intestinal permeability. These findings support a model in which *F. nucleatum*-derived succinic acid amplifies mucosal inflammation through macrophage-mediated inflammatory responses, accompanied by secondary impairment of epithelial and mucus barrier integrity. This process may create a feed-forward loop in which microbial dysbiosis, macrophage-driven inflammation, and barrier disruption reinforce one another during colitis.

These results should be interpreted in the context of the broader succinate literature. Succinate may have context-dependent effects depending on its cellular source, concentration, tissue compartment, receptor engagement, and inflammatory microenvironment. Succinate signaling appears to be context-dependent: while SUCNR1 activation has been reported to promote anti-inflammatory or tissue-protective programs in certain macrophage and neural stem cell contexts, studies in intestinal inflammation have shown that succinate–SUCNR1 signaling promotes colitis and fibrosis.^43^ Our data do not suggest that all succinate signaling is uniformly detrimental. Rather, they indicate that in the setting of *F. nucleatum* enrichment and ongoing intestinal inflammation, bacterial succinic acid functions as a pro-inflammatory effector that promotes macrophage activation through SUCNR1 and NF-κB signaling. This context-specific interpretation helps distinguish our findings from the broader literature on host-derived succinate metabolism.

These insights point to several therapeutic opportunities. Targeting *F. nucleatum* colonization, bacterial succinate production, or SUCNR1-dependent macrophage activation may represent potential strategies for attenuating inflammation in selected IBD patients with *F. nucleatum* enrichment. Inhibiting bacterial fumarate reductase activity could theoretically reduce succinic acid production by succinate-producing pathobionts, whereas pharmacological modulation of SUCNR1 may dampen downstream inflammatory signaling. However, given the pleiotropic roles of succinate in energy metabolism, tissue homeostasis, and host defense, systemic blockade of succinate signaling may carry unintended consequences.^39^ Future studies should explore local delivery, microbiota-selective interventions, and cell type-specific targeting strategies to define a therapeutic window.

This study has several limitations. First, biopsy sites could not be completely standardized across all patients because the anatomical distribution of active lesions differed between UC and CD patients. Although biopsies were preferentially obtained from endoscopically inflamed mucosal regions and disease extent/location information was recorded, regional variation in mucosal microbiota along the intestine may have contributed to inter-individual differences. Future studies with site-matched and paired inflamed/non-inflamed biopsies are needed to further validate these findings. Second, although our data support an important role of the succinic acid-SUCNR1 axis, the present siRNA-based in vitro approach cannot fully capture the complexity of SUCNR1-dependent and SUCNR1-independent signaling in vivo. In addition, the *frdA*-KO strain may exhibit metabolic adaptations beyond succinate depletion. Complementary genetic approaches, including macrophage-specific SUCNR1 deletion or conditional rescue, would further strengthen causal inference.

**Figure 11.**
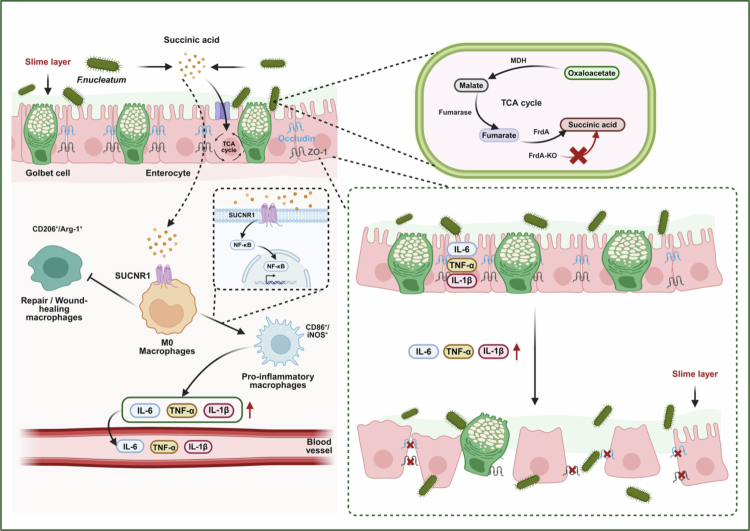
Schematic illustration of *F. nucleatum*-induced macrophage inflammatory activation and colitis exacerbation through the succinic acid–SUCNR1–NF-κB axis. *F. nucleatum* colonization increases succinic acid levels in the intestine and circulation. Succinic acid engages its cognate receptor, SUCNR1, on intestinal macrophages and activates downstream NF-κB signaling, thereby promoting pro-inflammatory macrophage activation characterized by increased expression of CD86 and iNOS and enhanced production of inflammatory cytokines, including IL-6, TNF-a, and IL-1ß. These macrophage-associated inflammatory responses are accompanied by epithelial barrier disruption, reduced goblet cell abundance and mucus barrier integrity, increased epithelial apoptosis, and aggravated mucosal and systemic inflammation. By contrast, the fumarate reductase-deficient (*frdA*-KO) *F. nucleatum* strain, which exhibits impaired succinic acid production, shows a reduced capacity to induce macrophage inflammatory activation or aggravate colitis. Exogenous succinic acid restores these effects, whereas SUCNR1 knockdown or NF-κB inhibition attenuates succinic acid-induced macrophage inflammatory activation. Together, these findings support a model in which *F. nucleatum* aggravates colitis through a macrophage-centered succinic acid–SUCNR1–NF-κB signaling axis.

In conclusion, this work identifies *F. nucleatum-*derived succinic acid as a microbial metabolite that promotes macrophage-mediated inflammation and epithelial barrier injury in experimental colitis. By linking *F. nucleatum* metabolism to SUCNR1-dependent NF-κB activation in macrophages, our findings extend the existing literature on succinate in IBD and clarify how a specific pathobiont-derived metabolite can amplify intestinal inflammation. The *F. nucleatum*–succinic acid–SUCNR1–NF-κB–macrophage axis may therefore represent a potential target for microbiota-directed or immunometabolic intervention in IBD (Figure 11).

## Supplementary Material

figureS3.png figureS3.png

figureS5.png figureS5.png

figureS8.png figureS8.png

supplementary materials_table.docx supplementary materials_table.docx

figureS2.png figureS2.png

figureS9.png figureS9.png

figureS4.png figureS4.png

figureS6.png figureS6.png

figureS10.png figureS10.png

supplementary materials2.xlsx supplementary materials2.xlsx

figureS11.png figureS11.png

figureS1.png figureS1.png

figureS7.png figureS7.png

Supplementary material KGMI_A_2702183_SM2174.docx

## Data Availability

Data are available on request from the corresponding author and will be deposited upon acceptance.

## References

[1] Christensen C , Knudsen A , Arnesen EK , Hatlebakk JG , Sletten IS , Fadnes LT . Diet, food, and nutritional exposures and inflammatory bowel disease or progression of disease: an umbrella review. Adv Nutr. 2024;15(5):100219. doi: 10.1016/j.advnut.2024.100219. 38599319 PMC11063602

[2] Wang S , Dong Z , Wan X . Global, regional, and national burden of inflammatory bowel disease and its associated anemia, 1990 to 2019 and predictions to 2050: an analysis of the global burden of disease study 2019. Autoimmun Rev. 2024;23(3):103498. doi: 10.1016/j.autrev.2023.103498. 38052263

[3] Brennan CA , Garrett WS . Fusobacterium nucleatum - symbiont, opportunist and oncobacterium. Nat Rev Microbiol. 2019;17(3):156–166. doi: 10.1038/s41579-018-0129-6. 30546113 PMC6589823

[4] Chen Y , Cao P , Su W , Zhan N , Dong W . Fusobacterium nucleatum facilitates ulcerative colitis through activating IL-17F signaling to NF-κB via the upregulation of CARD3 expression. J Pathol. 2020;250(2):170–182. doi: 10.1002/path.5358. 31610014

[5] Jiang SS , Chen YX , Fang JY . Fusobacterium nucleatum: ecology, pathogenesis and clinical implications. Nat Rev Microbiol. 2026;24(3):197–214. doi: 10.1038/s41579-025-01237-z. 40983729

[6] Gao X , Cao F , Li Y , Huang J , Hu X . Fusobacterium nucleatum: a transboundary pathogen in host-microbiota networks. Gut Pathog. 2025 Nov;17(1):92. doi: 10.1186/s13099-025-00775-4. 41261419 PMC12632115

[7] He Y , Fu L , Li Y , Wang W , Gong M , Zhang J , Dong X , Huang J , Mackay CR , Chen Y , et al. Gut microbial metabolites facilitate anticancer therapy efficacy by modulating cytotoxic CD8(+) T cell immunity. Cell Metab. 2021;33(5):988–1000.e7. doi: 10.1016/j.cmet.2021.03.002. 33761313

[8] Mager LF , Burkhard R , Pett N , Cooke NCA , Brown K , Ramay H , Paik S , Stagg J , Groves RA , Gallo M , et al. Microbiome-derived inosine modulates response to checkpoint inhibitor immunotherapy. Sci. 2020;369(6510):1481–1489. doi: 10.1126/science.abc3421. 32792462

[9] Ternes D , Tsenkova M , Pozdeev VI , Meyers M , Koncina E , Atatri S , Schmitz M , Karta J , Schmoetten M , Heinken A , et al. The gut microbial metabolite formate exacerbates colorectal cancer progression. Nat Metab. 2022;4(4):458–475. doi: 10.1038/s42255-022-00558-0. 35437333 PMC9046088

[10] Ooi M , Nishiumi S , Yoshie T , Shiomi Y , Kohashi M , Fukunaga K , Nakamura S , Matsumoto T , Hatano N , Shinohara M , et al. GC/MS-based profiling of amino acids and TCA cycle-related molecules in ulcerative colitis. Inflamm Res. 2011;60(9):831–40. doi: 10.1007/s00011-011-0340-7. 21523508

[11] Osaka T , Moriyama E , Arai S , Date Y , Yagi J , Kikuchi J , Tsuneda S . Meta-analysis of fecal microbiota and metabolites in experimental colitic mice during the inflammatory and healing phases. Nutrients. 2017;9(12):1329. doi: 10.3390/nu9121329. 29211010 PMC5748779

[12] Fremder M , Kim SW , Khamaysi A , Shimshilashvili L , Eini-Rider H , Park IS , Hadad U , Cheon JH , Ohana E . A transepithelial pathway delivers succinate to macrophages, thus perpetuating their pro-inflammatory metabolic state. Cell Rep. 2021;36(6):109521. doi: 10.1016/j.celrep.2021.109521. 34380041

[13] Fujiwara H , Seike K , Brooks MD , Mathew AV , Kovalenko I , Pal A , Lee H , Peltier D , Kim S , Liu C , et al. Mitochondrial complex II in intestinal epithelial cells regulates T cell-mediated immunopathology. Nat Immunol. 2021;22(11):1440–1451. doi: 10.1038/s41590-021-01048-3. 34686860 PMC9351914

[14] Zhao M , Zhou C , Wang D , Wu Q , Feng B . Succinate's dual roles in inflammatory bowel disease: a narrative review of microbiota-metabolism-immune crosstalk and therapeutic implications. Journal of inflammation research vol. 2025 Oct 28;18:15017–15032. doi: 10.2147/JIR.S561871. PMC12579456 41181356

[15] Hegarty LM , Jones G , Bain CC . Macrophages in intestinal homeostasis and inflammatory bowel disease. Nature reviews. Gastroenterology & hepatology. 2023;20(8):538–553. doi: 10.1038/s41575-023-00769-0. 37069320

[16] Zhou X , Li W , Wang S , Zhang P , Xiao J , Zheng X , Xu X , Xue S , Hui L , Ji H , et al. YAP aggravates inflammatory bowel disease by regulating M1/M2 macrophage polarization and gut microbial homeostasis. Cell Rep. 2019;27(4):1176–1189.e5. doi: 10.1016/j.celrep.2019.03.028. 31018132

[17] Zhao X , Di Q , Liu H , Quan J , Ling J , Xiao Y , Wu H , Song W , An H , Chen W . MEF2C promotes M1 macrophage polarization and Th1 responses. Cell Mol Immunol. 2022;19(4):540–553. doi: 10.1038/s41423-022-00841-w. 35194174 PMC8975968

[18] Hunter MM , Wang A , Parhar KS , Johnston MJ , Van Rooijen N , Beck PL , McKay DM . In vitro-derived alternatively activated macrophages reduce colonic inflammation in mice. Gastroenterology. 2010;138(4):1395–405. doi: 10.1053/j.gastro.2009.12.041. 20044996

[19] Zinger A , Choi D , Fear E , Fine Z , Cohen RD , Rubin DT . Long-term effectiveness and safety of risankizumab in patients with Crohn's disease. Clin Gastroenterol Hepatol. 2025;23(10):1817–1823. doi: 10.1016/j.cgh.2024.09.027. 39461462

[20] Rubin DT , Ananthakrishnan AN , Siegel CA , Barnes EL , Long MD . ACG clinical guideline update: ulcerative colitis in adults. Am J Gastroenterol. 2025;120(6):1187–1224. Published 2025 Jun 3.21. doi: 10.14309/ajg.0000000000003463. 40701556

[21] Turner D , Ricciuto A , Lewis A . STRIDE-II: an update on the selecting therapeutic targets in inflammatory bowel disease (STRIDE) initiative of the international organization for the study of IBD (IOIBD): determining therapeutic goals for treat-to-target strategies in IBD. Gastroenterology. 2021 Apr;160(5):1570–1583. doi: 10.1053/j.gastro.2020.12.031. 33359090

[22] Bolyen E , Rideout JR , Dillon MR , Bokulich NA , Abnet CC , Al-Ghalith GA , Alexander H , Alm EJ , Arumugam M , Asnicar F , et al. Reproducible, interactive, scalable and extensible microbiome data science using QIIME 2. Nat Biotechnol. 2019;37(8):852–857. doi: 10.1038/s41587-019-0209-9. 31341288 PMC7015180

[23] Quast C , Pruesse E , Yilmaz P , Gerken J , Schweer T , Yarza P , Peplies J , Glöckner FO . The SILVA ribosomal RNA gene database project: improved data processing and web-based tools. Nucleic Acids Res. 2013;41(Database issue):D590–D596. doi: 10.1093/nar/gks1219. 23193283 PMC3531112

[24] Chen Y , Zhang J , Cao P , Su W , Deng Y , Zhan N , Fu X , Huang Y , Dong W . Fusobacterium nucleatum promotes metastasis in colorectal cancer by activating autophagy signaling via the upregulation of CARD3 expression. Theranostics. 2020;10(1):323–339. doi: 10.7150/thno.38870. 31903123 PMC6929621

[25] Xiang Z , Li X , Wang X , Deng B , He H , Xu M , Wu X , Tan C , Liu Y , Yu B , et al. Fusobacterium nucleatum exacerbates colitis via STAT3 activation induced by Acetyl-CoA accumulation. Gut Microbes. 2025;17(1):2489070. doi: 10.1080/19490976.2025.2489070. 40212016 PMC12931693

[26] Yu T , Guo F , Sun T , Ma D , Han J , Qian Y , Kryczek I , Nagarsheth N , Chen Y , Hong J , et al. Fusobacterium nucleatum promotes chemoresistance to colorectal cancer by modulating autophagy. Cell. 2017;170(3):548–563.e16. doi: 10.1016/j.cell.2017.07.008. 28753429 PMC5767127

[27] Jiang SS , Xie Y , Xiao X , Kang Z , Lin X , Zhang L , Li C , Qian Y , Xu P , Leng X , et al. Fusobacterium nucleatum-derived succinic acid induces tumor resistance to immunotherapy in colorectal cancer. Cell Host Microbe. 2023;31(5):781–797.e9. doi: 10.1016/j.chom.2023.04.010. 37130518

[28] Wu J , Wei Z , Cheng P , Qian C , Xu F , Yang Y , Wang A , Chen W , Sun Z , Lu Y . Rhein modulates host purine metabolism in intestine through gut microbiota and ameliorates experimental colitis. Theranostics. 2020;10(23):10665–10679. Published 2020 Aug 29. doi: 10.7150/thno.43528. 32929373 PMC7482825

[29] Li W , Liu Y , Zheng X , Han J , Shi A , Wong CC , Wang R , Jing X , Fan S , Zhang C , et al. Rewiring tryptophan metabolism via programmable probiotic integrated by dual-layered microcapsule protects against inflammatory bowel disease in mice. ACS Nano. 2024;18(52):35443–35464. doi: 10.1021/acsnano.4c12801. 39609102

[30] Li Y , Yuan R , Wang Y , Guo C . Bifidobacterium breve-derived indole-3-lactic acid ameliorates colitis-associated tumorigenesis by directing the differentiation of immature colonic macrophages. Theranostics. 2024;14(7):2719–2735. doi: 10.7150/thno.92350. 38773969 PMC11103503

[31] Cao P , Chen Y , Guo X , Su W , Zhan N , Dong W . Fusobacterium nucleatum activates endoplasmic reticulum stress to promote Crohn's disease development via the upregulation of CARD3 expression. Front Pharmacol. 2020;11:106. doi: 10.3389/fphar.2020.00106. 32153411 PMC7047714

[32] Zhu W , Yu J , Nie Y , Shi X , Liu Y , Li F , Zhang X . Disequilibrium of M1 and M2 macrophages correlates with the development of experimental inflammatory bowel diseases. Immunol Invest. 2014;43(7):638–52. doi: 10.3109/08820139.2014.909456. 24921428

[33] Martin-Gallausiaux C , Salesse L , Garcia-Weber D , Marinelli L , Beguet-Crespel F , Brochard V , Le Gléau C , Jamet A , Doré J , Blottière HM , et al. Fusobacterium nucleatum promotes inflammatory and anti-apoptotic responses in colorectal cancer cells via ADP-heptose release and ALPK1/TIFA axis activation. Gut Microbes. 2024;16(1):2295384. doi: 10.1080/19490976.2023.2295384. 38126163 PMC10761154

[34] Ning L , Zhou Y , Sun H , Zhang Y , Shen C , Wang Z , Xuan B , Zhao Y , Ma Y , Yan Y , et al. Microbiome and metabolome features in inflammatory bowel disease via multi-omics integration analyses across cohorts. Nat Commun. 2023;14(1):7135. doi: 10.1038/s41467-023-42788-0. 37932270 PMC10628233

[35] Fernández-Veledo S , Grau-Bové C , Notararigo S , Huber-Ruano I . The role of microbial succinate in the pathophysiology of inflammatory bowel disease: mechanisms and therapeutic potential. Curr Opin Microbiol. 2025;85:102599. doi: 10.1016/j.mib.2025.102599. 40132355

[36] McDonald NC , White RL . Reduction of fumarate to succinate mediated by fusobacterium varium. Appl Biochem Biotechnol. 2019;187(1):163–175. doi: 10.1007/s12010-018-2817-0. 29911265

[37] Zhao Y , Ning L , Yan Y , Ding J , Yu B , Yang P , Shen N , Xuan B , Wang Z , Zhang Y , et al. Gut pathogen clostridium symbiosum rewires macrophage succinylation to drive enteric neuron loss in inflammatory bowel disease. Cell Host Microbe. 2026;34(4):620–638.e10. doi: 10.1016/j.chom.2026.03.001. 41881016

[38] He W , Miao FJ , Lin DC , Schwandner RT , Wang Z , Gao J , Chen J , Tian H , Ling L . Citric acid cycle intermediates as ligands for orphan G-protein-coupled receptors. Natur. 2004;429(6988):188–193. doi: 10.1038/nature02488. 15141213

[39] Macias-Ceja DC , Ortiz-Masiá D , Salvador P , Gisbert-Ferrándiz L , Hernández C , Hausmann M , Rogler G , Esplugues JV , Hinojosa J , Alós R , et al. Succinate receptor mediates intestinal inflammation and fibrosis. Mucosal Immunol. 2019;12(1):178–187. doi: 10.1038/s41385-018-0087-3. 30279517

[40] Connors J , Dawe N , Van Limbergen J . The role of succinate in the regulation of intestinal inflammation. Nutrients. 2018;11(1):25. doi: 10.3390/nu11010025. 30583500 PMC6356305

[41] Huang H , Li G , He Y , Chen J , Yan J , Zhang Q , Cai X . Cellular succinate metabolism and signaling in inflammation: implications for therapeutic intervention. Front Immunol. 2024;15:1404441. doi: 10.3389/fimmu.2024.1404441. 38933270 PMC11200920

[42] Liu H , Hong XL , Sun TT , Huang XW , Wang JL , Xiong H . Fusobacterium nucleatum exacerbates colitis by damaging epithelial barriers and inducing aberrant inflammation. J Dig Dis. 2020;21(7):385–398. doi: 10.1111/1751-2980.12909. 32441482

[43] Peruzzotti-Jametti L , Bernstock JD , Vicario N , Costa AS , Kwok CK , Leonardi T , Booty LM , Bicci I , Balzarotti B , Volpe G , et al. Macrophage-derived extracellular succinate licenses neural stem cells to suppress chronic neuroinflammation. Cell Stem Cell. 2018;22(3):355–368.e13. doi: 10.1016/j.stem.2018.01.020. 29478844 PMC5842147

