# Supplementary figures and images for: Fusobacterium nucleatum-derived succinic acid aggravates colitis by triggering macrophage pro-inflammatory phenotypic transformation via SUCNR1/NF-κB axis
Source: Gut Microbes. 2026 Jul 15;18(1):2702183. doi: 10.1080/19490976.2026.2702183 (PMC13374752; doi:10.1080/19490976.2026.2702183)

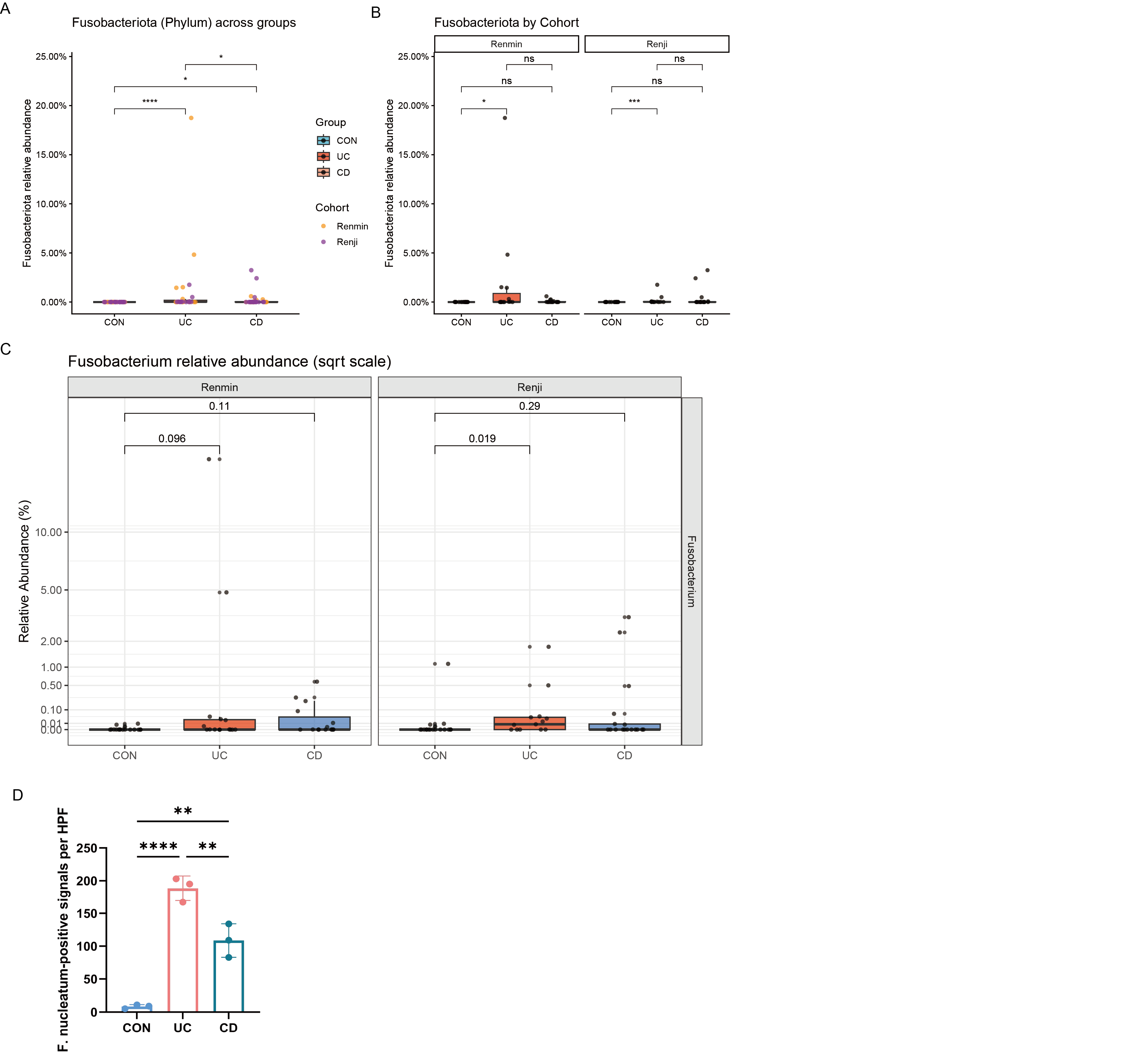

Supplement: figureS3.png [file KGMI_A_2702183_SM3621.png]

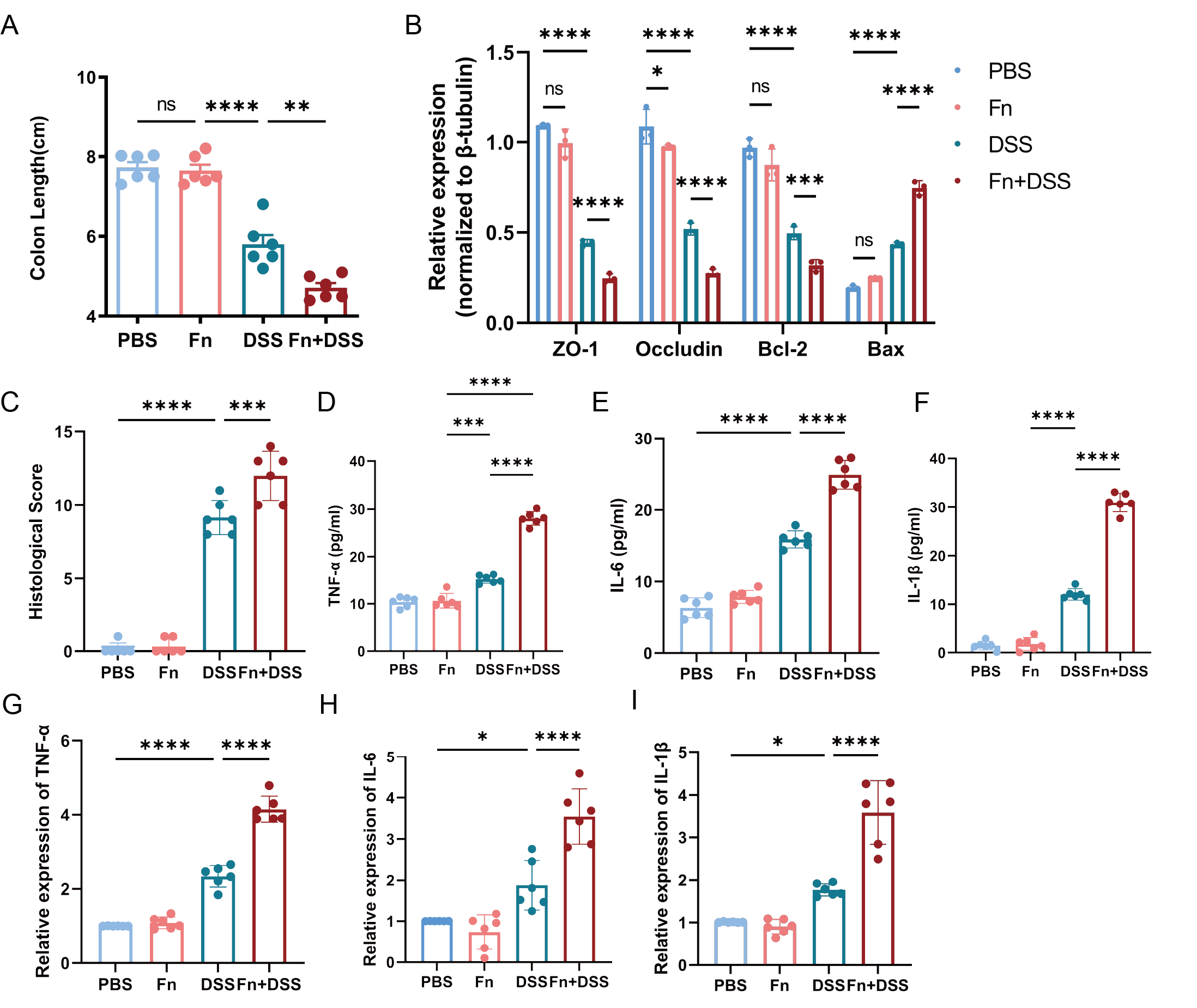

Supplement: figureS5.png [file KGMI_A_2702183_SM3622.png]

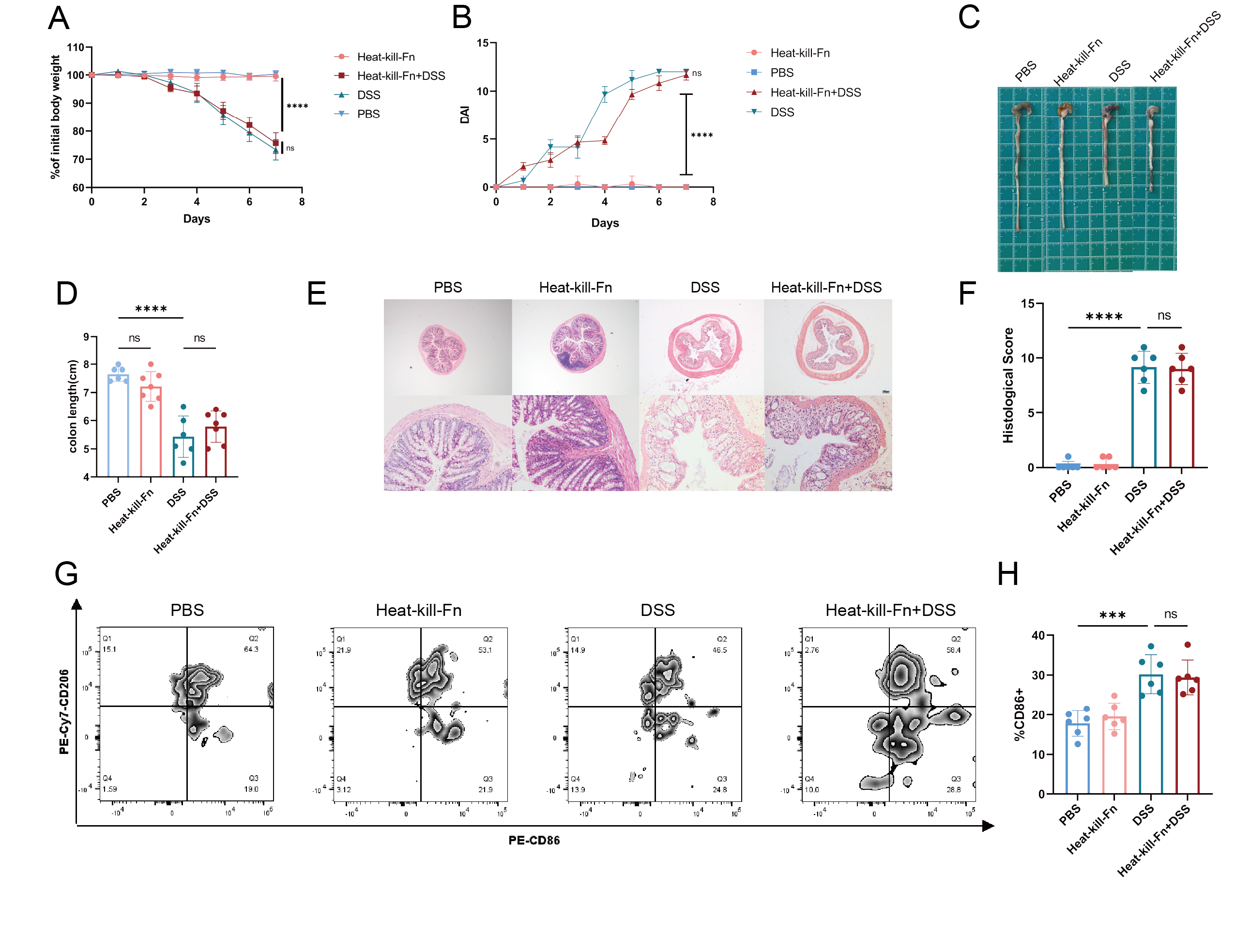

Supplement: figureS8.png [file KGMI_A_2702183_SM3623.png]

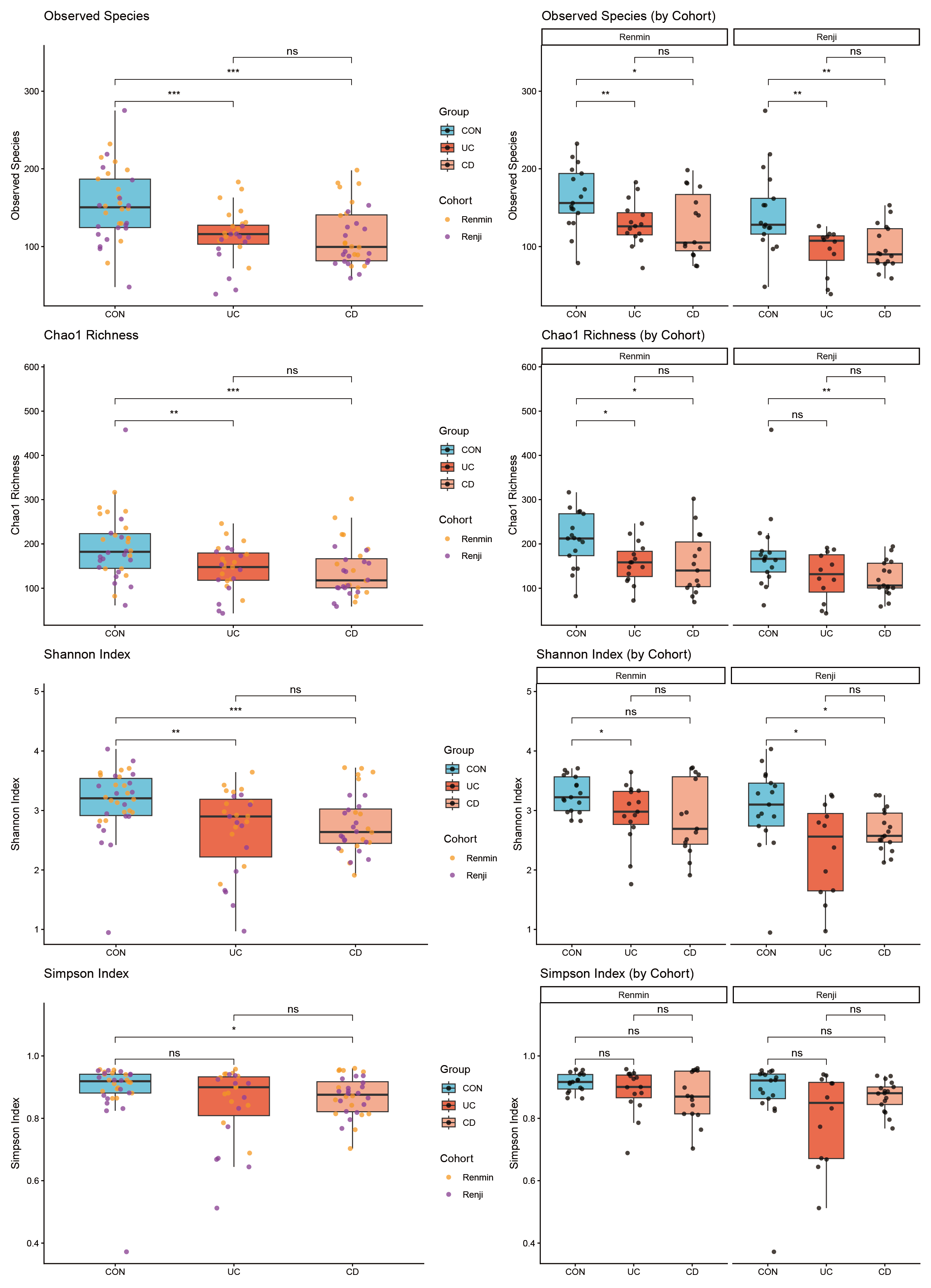

Supplement: figureS2.png [file KGMI_A_2702183_SM3625.png]

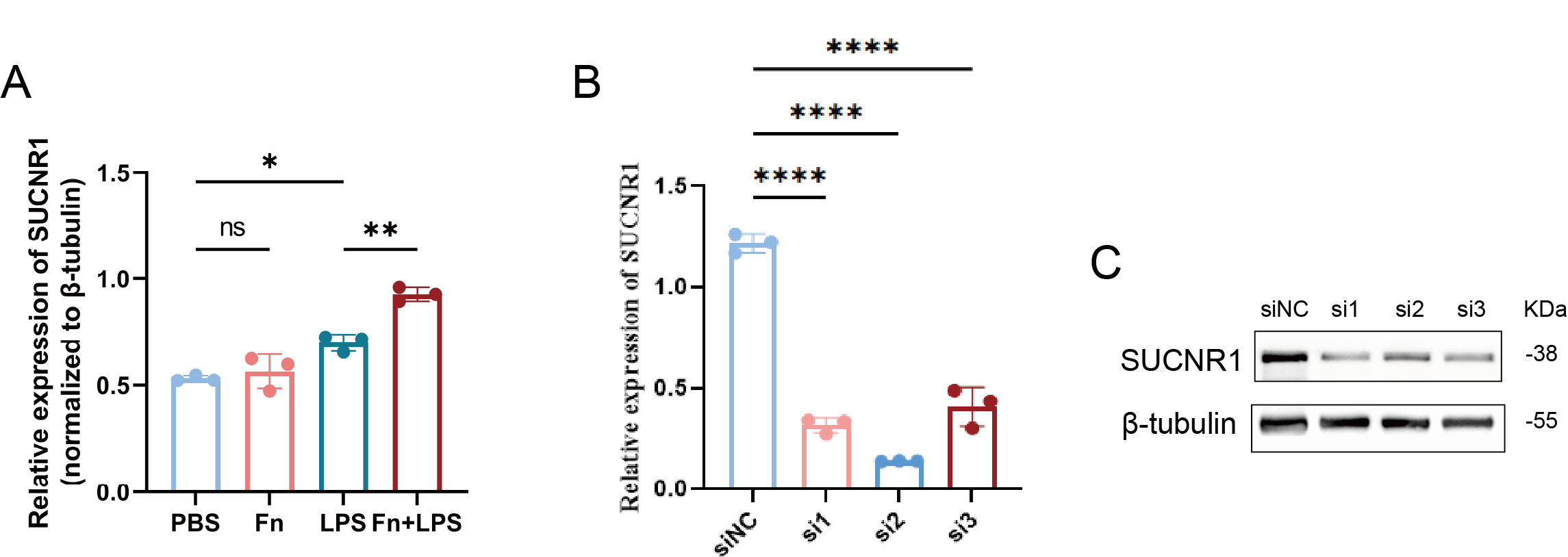

Supplement: figureS9.png [file KGMI_A_2702183_SM3626.png]

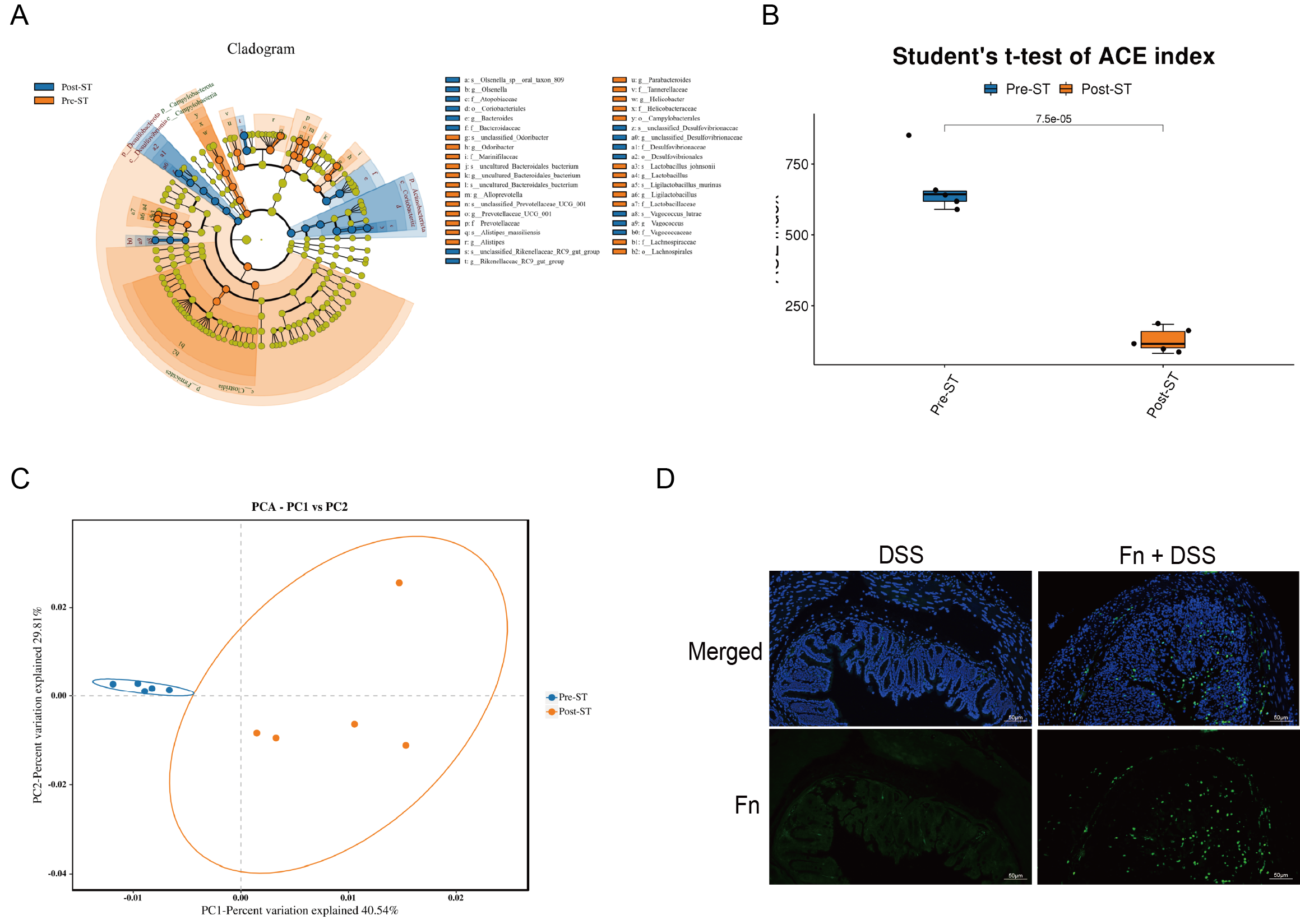

Supplement: figureS4.png [file KGMI_A_2702183_SM3627.png]

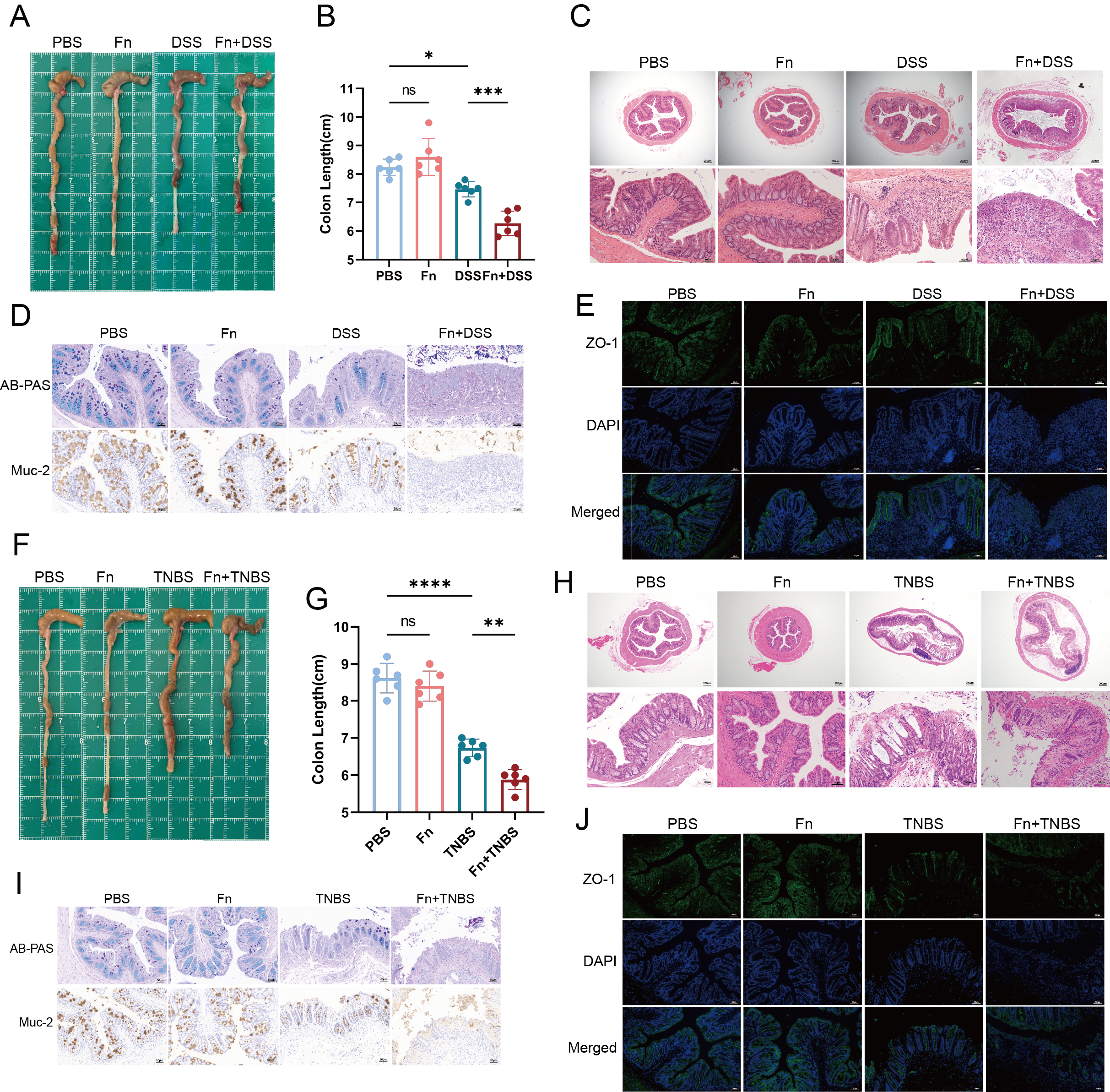

Supplement: figureS6.png [file KGMI_A_2702183_SM3628.png]

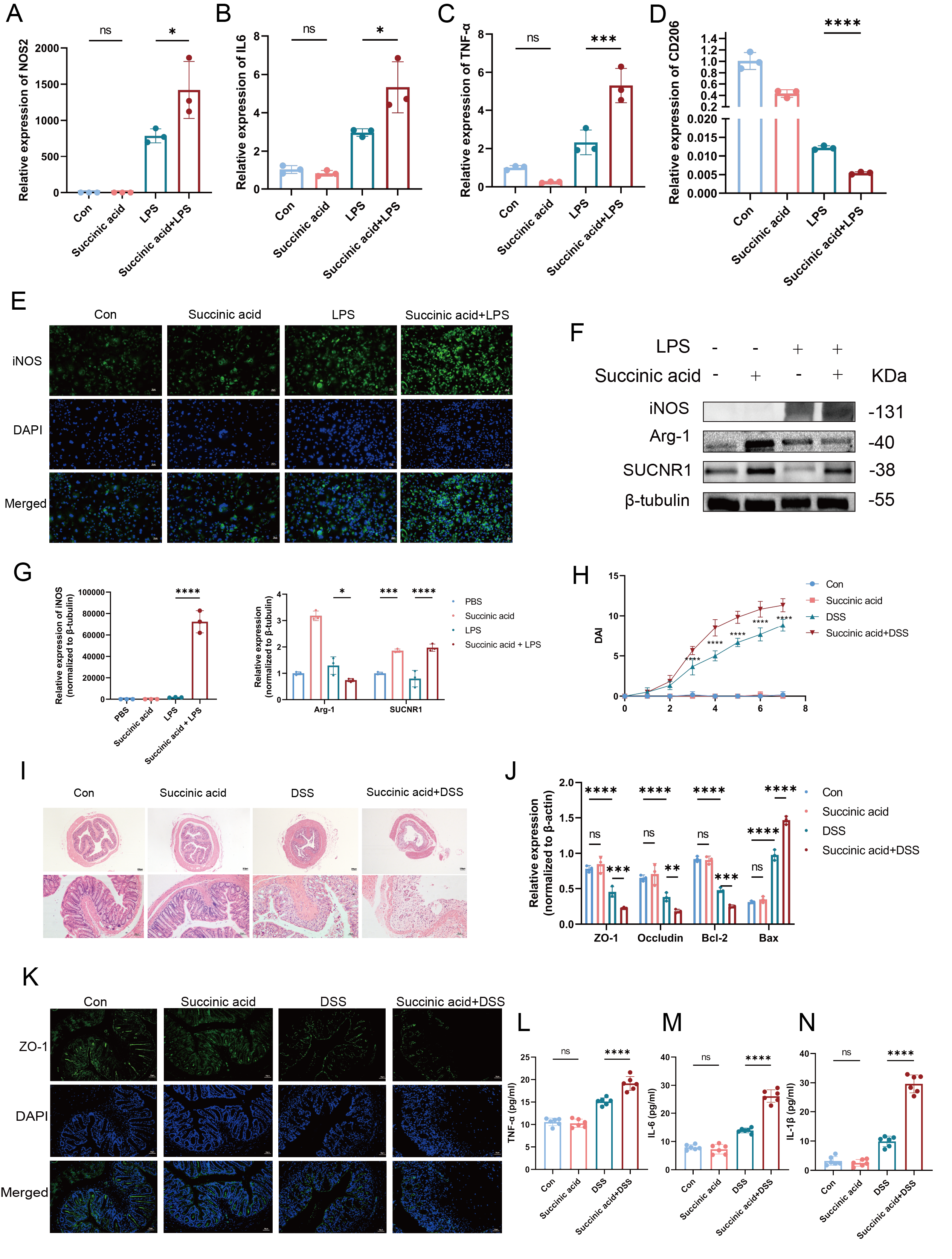

Supplement: figureS10.png [file KGMI_A_2702183_SM3629.png]

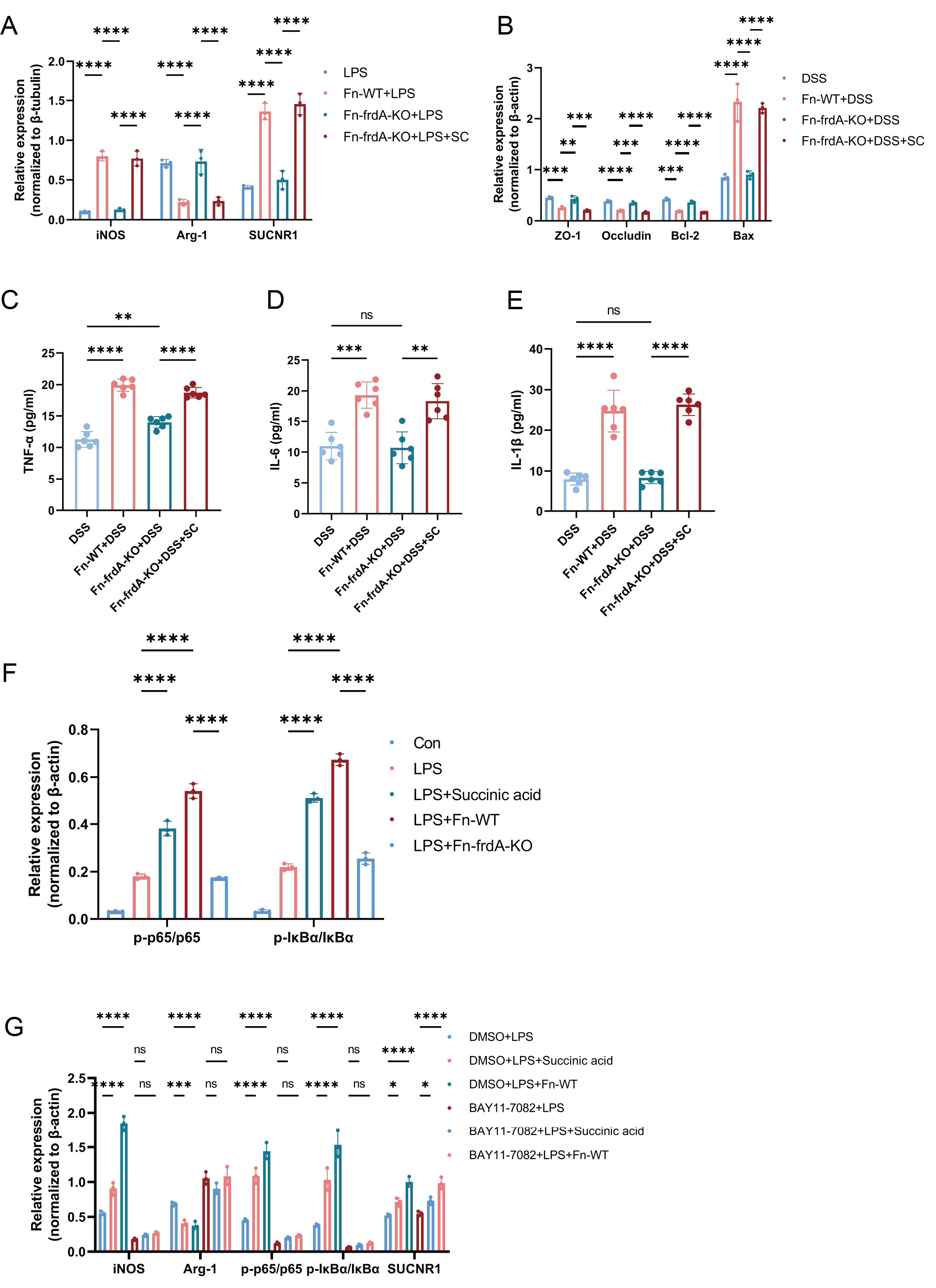

Supplement: figureS11.png [file KGMI_A_2702183_SM3631.png]

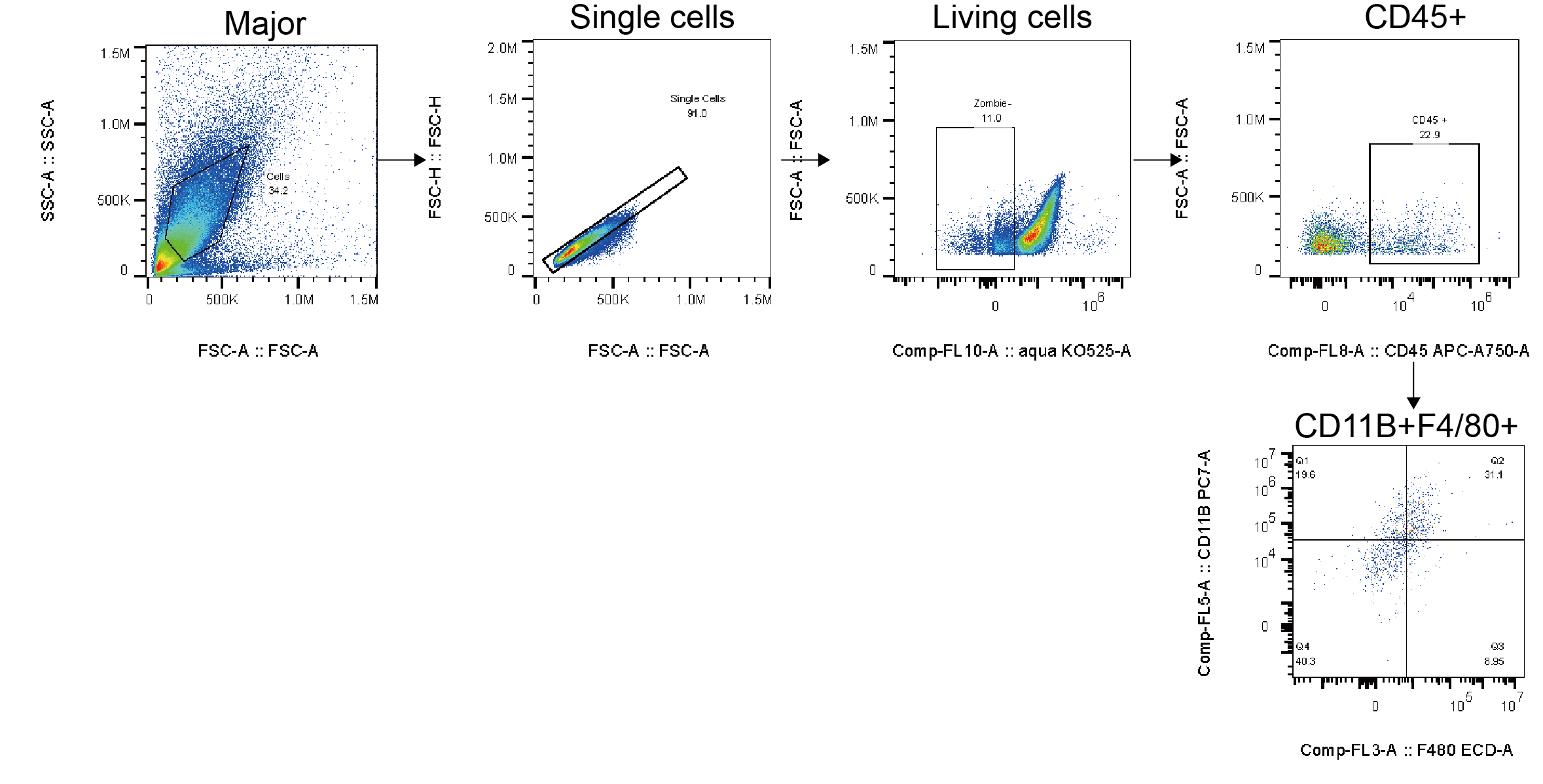

Supplement: figureS1.png [file KGMI_A_2702183_SM3632.png]

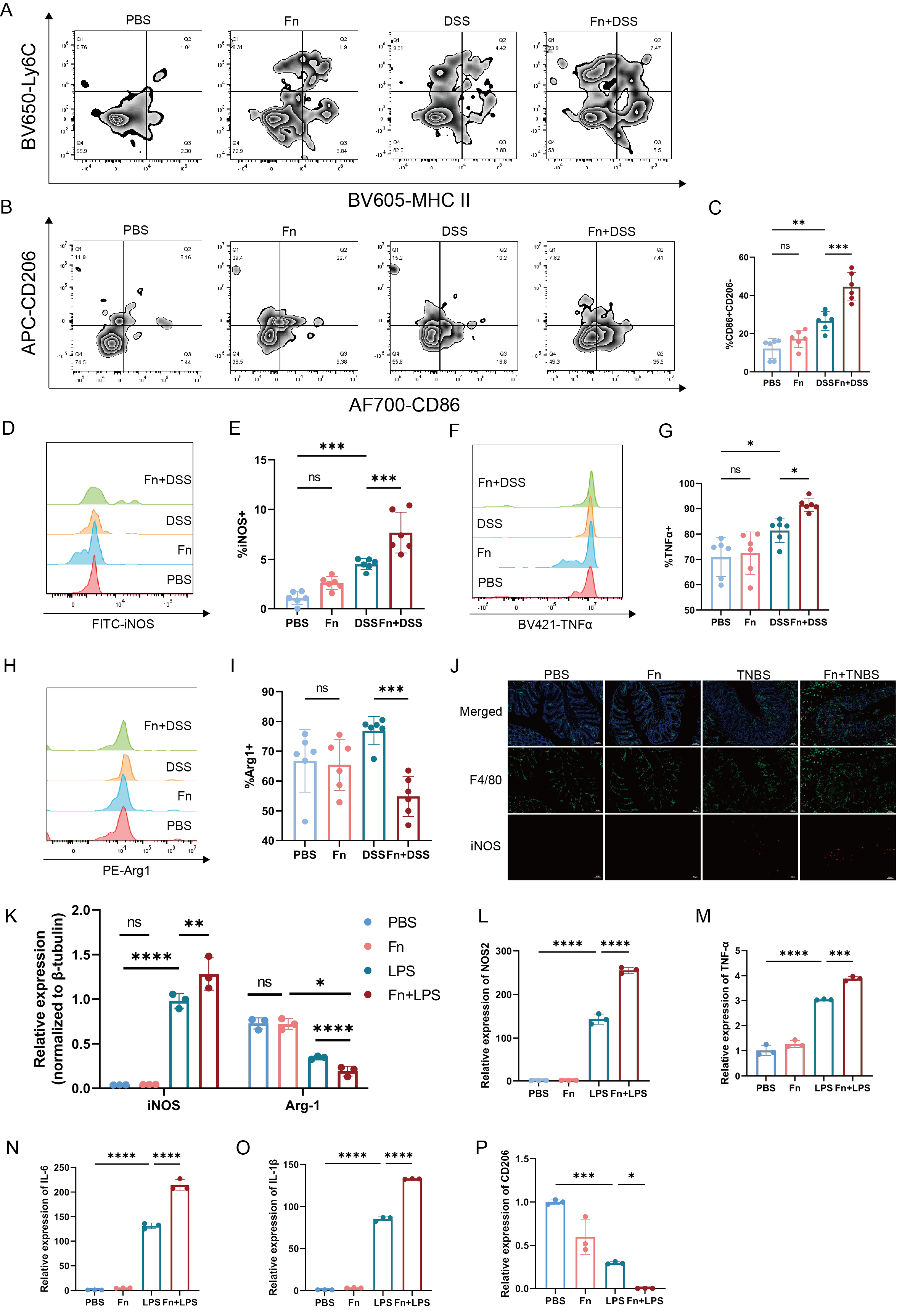

Supplement: figureS7.png [file KGMI_A_2702183_SM3633.png]
