# Supplementary material for: Fusobacterium nucleatum-derived succinic acid aggravates colitis by triggering macrophage pro-inflammatory phenotypic transformation via SUCNR1/NF-κB axis
Source: Gut Microbes. 2026 Jul 15;18(1):2702183. doi: 10.1080/19490976.2026.2702183 (PMC13374752; doi:10.1080/19490976.2026.2702183)
Supplement: supplementary materials_table.docx [file KGMI_A_2702183_SM3624.docx]

**Table S1.** Clinical characteristics of participants in the Renmin fecal microbiota cohort

|  | CON (n=17) | UC  (n=15) | CD  (n=15) | *p* value |
| --- | --- | --- | --- | --- |
| Age, years | 37.00 ± 14.53 | 35.53 ± 14.63 | 30.87 ± 16.00 | 0.499 |
| Gender |  |  |  | 0.668 |
| Male n (%) | 7(41.2) | 8(53.3) | 9(60.0) |  |
| Female n (%) | 10(58.8) | 7(46.7) | 6(40.0) |  |

**Table S2.** Clinical characteristics of participants in the Renji fecal microbiota cohort

|  | CON (n=17) | UC  (n=12) | CD  (n=17) | *p* value |
| --- | --- | --- | --- | --- |
| Age, years | 42.18 ± 14.33 | 40.00 ± 15.12 | 35.24 ± 12.08 | 0.335 |
| Gender |  |  |  | 0.920 |
| Male n (%) | 11 (64.7) | 7 (58.3) | 10 (58.8) |  |
| Female n (%) | 6 (35.3) | 5 (41.7) | 7 (41.2) |  |

**Table S3.** Clinical and sampling characteristics of participants included in mucosal 16S rRNA sequencing

|  | UC  (n=11) | CD  (n=6) | *p* value |
| --- | --- | --- | --- |
| Age, years | 41.73 ± 7.38 | 36.83 ± 12.51 | 0.409 |
| Gender |  |  |  |
| Male n (%) | 4 (36.4) | 3 (50.0) | 0.644 |
| Female n (%) | 7 (63.6) | 3 (50.0) |  |
| Disease activity grade |  |  | 0.467 |
| Mild, n (%) | 2 (18.2) | 0 (0.0) |  |
| Moderate, n (%) | 6 (54.5) | 5 (83.3) |  |
| Severe, n (%) | 3 (27.3) | 1 (16.7) |  |
| Disease extent/location |  |  |  |
| Rectum, n (%) | 1 (9.1) |  |  |
| Left side, n (%) | 5 (45.5) |  |  |
| Total colitis n (%) | 5 (45.5) |  |  |
| L1, n (%) |  | 2 (33.3) |  |
| L2, n (%) |  | 1 (16.7) |  |
| L3, n (%) |  | 3 (50.0) |  |

L1, ileal disease; L2, colonic disease; L3, ileocolonic disease.

**Table S4.** Clinical and sampling characteristics of participants included in mucosal *F. nucleatum* qPCR analysis

|  | CON (n=14) | UC  (n=10) | CD  (n=13) | *p* value |
| --- | --- | --- | --- | --- |
| Age, years | 37.64 ± 13.22 | 38.60 ± 11.78 | 34.92 ± 13.98 | 0.778 |
| Gender |  |  |  | 0.071 |
| Male n (%) | 5 (35.7) | 4 (40.0) | 10 (76.9) |  |
| Female n (%) | 9 (64.3) | 6 (60.0) | 3 (23.1) |  |
| Disease activity |  |  |  | 0.103 |
| Mild, n (%) |  | 3 (30.0) | 0 (0.0) |  |
| Moderate, n (%) |  | 5 (50.0) | 10 (76.9) |  |
| Severe, n (%) |  | 2 (20.0) | 3 (23.1) |  |
| Disease extent/location |  |  |  |  |
| Rectum, n (%) |  | 3 (30.0) |  |  |
| Left side, n (%) |  | 4 (40.0) |  |  |
| Total colitis n (%) |  | 3 (30.0) |  |  |
| L1, n (%) |  |  | 5 (38.5) |  |
| L2, n (%) |  |  | 2 (15.4) |  |
| L3, n (%) |  |  | 5 (38.5) |  |
| L4, n (%) |  |  | 1 (7.7) |  |

L1, ileal disease; L2, colonic disease; L3, ileocolonic disease; L4, upper gastrointestinal involvement.

**Table S5.** Clinicopathological characteristics of participants included in the FISH cohort

|  | CON (n=10) | UC  (n=60) | CD  (n=41) | *p* value |
| --- | --- | --- | --- | --- |
| Age, years | 39.10 ± 14.91 | 45.82 ± 13.85 | 34.78 ± 13.33 | 0.001 |
| Gender |  |  |  | 0.448 |
| Male n (%) | 5 (50.0) | 35 (58.3) | 28 (68.3) |  |
| Female n (%) | 5 (50.0) | 25 (41.7) | 13 (31.7) |  |
| Clinical course |  |  |  | 0.270 |
| Remission, n (%) |  | 12 (20.0) | 13 (31.7) |  |
| Active, n (%) |  | 48 (80.0) | 28 (68.3) |  |
| Disease activity |  |  |  | 0.037 |
| Mild, n (%) |  | 17 (28.3) | 0 (0.0) |  |
| Moderate, n (%) |  | 19 (31.7) | 24 (58.5) |  |
| Severe, n (%) |  | 12 (20.0) | 4 (9.8) |  |
| Disease extent/location |  |  |  |  |
| Rectum, n (%) |  | 8 (13.3) |  |  |
| Left side, n (%) |  | 27 (45.0) |  |  |
| Total colitis n (%) |  | 25 (41.7) |  |  |
| L1, n (%) |  |  | 15 (36.6) |  |
| L2, n (%) |  |  | 8 (19.5) |  |
| L3, n (%) |  |  | 17 (41.5) |  |
| L4, n (%) |  |  | 1 (2.4) |  |

L1, ileal disease; L2, colonic disease; L3, ileocolonic disease. L4, upper gastrointestinal involvement.

**Table S6.** Scoring Scale of Disease Activity Index (DAI).

| Score | weight decrease (%) | Stool consistency | Rectal bleeding |
| --- | --- | --- | --- |
| 0 | no loss | normal | no blood |
| 1 | 1-5 | loose stool |  |
| 2 | 5-10 | watery diarrhea | Presence of blood |
| 3 | 10-20 | slimy diarrhea, little blood |  |
| 4 | ＞20 | severe watery diarrhea with blood | gross bleeding |

**Table S7.** Histopathological scoring system.

| Score | Extent of damage | Decreased mucus in glands | Tissue Damage | Inflammatory Cell Infiltration |
| --- | --- | --- | --- | --- |
| 0 | 0 | None | No mucosal damage | Few inflammatory cells in the lamina propria |
| 1 | ≤25% | Mild | Discrete epithelial lesions | Increased number of inflammatory cells in the lamina propria |
| 2 | ≤50% | Moderate | Surface mucosal erosion or focal ulceration | Aggregated inflammatory cells extending to the submucosa |
| 3 | ≤75% | Moderate | Extensive mucosal injury with extension to  deeper structures of the intestinal wall | Transmural extension of infiltrates |
| 4 | ≤100% | Severe | - | - |

**Table S8.** Primer sequences utilized for quantitative polymerase chain reaction (qPCR)

| Target | | Sequence 5’-3’ |
| --- | --- | --- |
| m-β-actin | sense | GGCTGTATTCCCCTCCATCG |
|  | antisense | CCAGTTGGTAACAATGCCATGT |
| m-NOS2 | sense | GGAGTGACGGCAAACATGACT |
|  | antisense | TCGATGCACAACTGGGTGAAC |
| m-IL-10 | sense | GCTGGACAACATACTGCTAACC |
|  | antisense | ATTTCCGATAAGGCTTGGCAA |
| m-IL-1β | sense | ATGATGGCTTATTACAGTGGCAA |
|  | antisense | GTCGGAGATTCGTAGCTGGA |
| m-CD206 | sense | CTCTGTTCAGCTATTGGACGC |
|  | antisense | CGGAATTTCTGGGATTCAGCTTC |
| m-SUCNR1 | sense | TCTTGTGAGAATTGGTTGGCAA |
|  | antisense | CATCTCCATAGGTCCCCTTATCA |
| m-IL-6 | sense | AGGTCGGTGTGAACGGATTTG |
|  | antisense | TGTAGACCATGTAGTTGAGGTCA |
| m-TNF-α | sense | CAGGCGGTGCCTATGTCTC |
|  | antisense | CGATCACCCCGAAGTTCAGTAG |
| m-IL-1β | sense | GAAATGCCACCTTTTGACAGTG |
|  | antisense | TGGATGCTCTCATCAGGACAG |

**Table S9.** siRNA sequence

| Target | | Sequence 5’-3’ |
| --- | --- | --- |
| m-siSUCNR1-1 | Forward | GGAACAGCAGCAAUGUCUA/dT//dT/ |
|  | Reverse | UAGACAUUGCUGCUGUUCC/dT//dT/ |
| m-siSUCNR1-2 | Forward | GGAGAUGUUCUCUGUAUAA/dT//dT/ |
|  | Reverse | UUAUACAGAGAACAUCUCC/dT//dT/ |
| m-siSUCNR1-3 | Forward | AGAUGGUAGUCUUCUUAAA/dT//dT/ |
|  | Reverse | UUUAAGAAGACUACCAUCU/dT//dT/ |
| m-siNC | Forward | UUC UCC GAA CGU GUC ACG UTT |
|  | Reverse | ACG UGA CAC GUU CGG AGA ATT |

**Table S10.** Association between *F. nucleatum* abundance and clinical phenotypes in patients with UC

|  | Fn-high (n=31) | Fn-negative/low (n=29) | *p* value |
| --- | --- | --- | --- |
| Gender |  |  | 0.570 |
| Male, n (%) | 17 (54.8) | 18 (62.1) |  |
| Female, n (%) | 14 (45.2) | 11 (37.9) |  |
| Age |  |  | 0.361 |
| ≤40, n (%) | 12 (38.7) | 8 (27.6) |  |
| >40, n (%) | 19 (61.3) | 21 (72.4) |  |
| Location |  |  | 0.381 |
| Rectum, n (%) | 5 (16.1) | 3 (10.3) |  |
| Left side, n (%) | 11 (35.5) | 16 (55.2) |  |
| Total colitis, n (%) | 15 (48.4) | 10 (34.5) |  |
| Clinical course |  |  | 0.272 |
| Active, n (%) | 27 (87.1) | 21 (72.4) |  |
| Remission, n (%) | 4 (12.9) | 8 (27.6) |  |
| Clinical activity |  |  | 0.010* |
| Mild, n (%) | 5 (18.5) | 12 (57.1) |  |
| Moderate, n (%) | 12 (44.4) | 7 (33.3) |  |
| Severe, n (%) | 10 (37.0) | 2 (9.5) |  |
| Refractory |  |  | 0.021* |
| Yes, n (%) | 9 (29.0) | 1 (3.4) |  |
| No, n (%) | 22 (71.0) | 28 (96.6) |  |

Associations with *p* values < 0.05 were indicated by *

|  | *Fn*-high (n=23) | | | *Fn*-negative/low (n=18) | | | | *p* value | |  |
| --- | --- | --- | --- | --- | --- | --- | --- | --- | --- | --- |
| Gender | |  |  | |  |  | | | 0.024* | |
| Male, n (%) | 17 (73.9) | | 7 (38.9) | | | |  | | |  |
| Female, n (%) | 6 (26.1) | | 11 (61.1) | | | |  |  |  |  |
| Age |  | |  | | | | 0.586 | | |  |
| ≤40, n (%) | 15 (65.2) | | 13 (72.2) | | | |  | | |  |
| >40, n (%) | 8 (34.8) | | 5 (27.8) | | | |  |  |  |  |
| Clinical course | | |  | | | | 0.082 | | |  |
| Moderate, n (%) | 16 (69.6) | | 8 (44.4) | | | |  | | |  |
| Severe, n (%) | 3 (13.3) | | 1 (5.6) | | | |  |  |  |  |
| Remission, n (%) | 4 (17.4) | | 9 (50.0) | | | |  |  |  |  |
| Location |  | |  | | | | 0.588 | | |  |
| L1, n (%) | 9 (39.1) | | 6 (33.3) | | | |  | | |  |
| L2, n (%) | 3 (13.0) | | 5 (27.8) | | | |  | | |  |
| L3, n (%) | 10 (43.5) | | 7 (38.9) | | | |  |  |  |  |
| L4, n (%) | 1 (4.3) | | 0 (0.0) | | | |  | | |  |
| Behavior |  | |  | | | | 0.153 | | |  |
| B1, n (%) | 5 (21.7) | | 9 (50.0) | | | |  | | |  |
| B2, n (%) | 10 (43.5) | | 6 (33.3) | | | |  | | |  |
| B3, n (%) | 8 (34.8) | | 3 (16.7) | | | |  | | |  |
| Perianal disease | | |  | | | | 0.045* | | |  |
| Yes, n (%) | 5 (21.7) | | 0 (0.0) | | | |  | | |  |
| No, n (%) | 18 (78.3) | | 18 (100.0) | | | |  |  |  |  |
| Surgery history |  | |  | | | | 0.044* | | |  |
| Yes, n (%) | 12 (52.2) | | 3 (16.7) | | | |  | | |  |
| No, n (%) | 11 (47.8) | | 15 (83.3) | | | |  |  |  |  |

**Table S11.** Association between *F. nucleatum* abundance and clinical phenotypes in patients with CD

Surgery history included prior intestinal resection or perianal surgery documented in the medical records. No included patient had a history of ileocecal valve removal. L1, ileal disease; L2, colonic disease; L3, ileocolonic disease; L4, upper gastrointestinal involvement; B1, non-stricturing non-penetrating; B2, stricturing; B3, penetrating. Associations with *p* values < 0.05 were indicated by *
