# Supplementary material for: Fusobacterium nucleatum-derived succinic acid aggravates colitis by triggering macrophage pro-inflammatory phenotypic transformation via SUCNR1/NF-κB axis
Source: Gut Microbes. 2026 Jul 15;18(1):2702183. doi: 10.1080/19490976.2026.2702183 (PMC13374752; doi:10.1080/19490976.2026.2702183)
Supplement: Supplementary material — KGMI_A_2702183_SM2174.docx [file KGMI_A_2702183_SM2174.docx]

**Figure S1.** **Gating strategy for the identification of intestinal macrophages by flow cytometry.**

Representative flow cytometry plots showing the gating strategy used to identify intestinal macrophages from colonic lamina propria single-cell suspensions. Cells were first gated based on FSC-A and SSC-A to exclude debris, followed by singlet selection using FSC-A and FSC-H. Living cells were identified as Zombie Aqua-negative cells, and leukocytes were then gated as CD45⁺ cells. Intestinal macrophages were subsequently defined as CD45⁺CD11b⁺F4/80⁺ cells for downstream phenotypic analysis.

**Figure S2. Alpha diversity of fecal microbiota in patients with IBD and healthy controls.**
Alpha diversity of fecal microbiota was assessed using Observed species, Chao1 richness, Shannon index, and Simpson index in healthy controls (CON), UC patients, and CD patients from the Renmin and Renji cohorts. Left panels show the pooled analysis of both cohorts, and right panels show cohort-stratified comparisons. Data are presented as box plots with individual data points. *P < 0.05, **P < 0.01, ***P < 0.001*;* ns, not significant.

**Figure S3.**

(A) Pooled analysis of *Fusobacteriota* abundance across the Renmin and Renji cohorts. (B) Cohort-stratified analysis of *Fusobacteriota* abundance. (C) Cohort-stratified analysis of Fusobacterium abundance shown on a square-root-transformed scale. (D) Representative field-based quantification of FUS664-positive F. nucleatum signals per high-power field (HPF) in control, UC, and CD mucosal tissues. Three representative HPFs were quantified for each group. Data are presented as box plots with individual data points. *P < 0.05, **P < 0.001, ***P < 0.0001; ns, not significant.

**Figure S4. Streptomycin pretreatment reshapes the endogenous gut microbiota and facilitates *F. nucleatum* colonization.**

(A) LEfSe cladogram showing differentially enriched bacterial taxa in fecal samples before and after 3 days of streptomycin treatment. Pre-ST, before streptomycin treatment; Post-ST, after streptomycin treatment. (B) Alpha diversity analysis of fecal microbiota before and after streptomycin treatment, as assessed by the ACE index. (C) Beta diversity analysis showing separation of fecal microbial communities before and after streptomycin treatment. (D) Representative fluorescence images confirming intestinal colonization of F. nucleatum after gavage in DSS-treated mice. Data are presented as mean ± SEM.

**Figure S5. *F. nucleatum* exacerbates DSS-induced colitis and enhances inflammatory responses in mice.**

(A) Quantification of colon length in the acute DSS-induced colitis model. (B) Quantification of Western blot analysis showing the relative expression of tight junction proteins ZO-1 and occludin, the anti-apoptotic protein Bcl-2, and the pro-apoptotic protein Bax in colonic tissues. Protein levels were normalized to β-tubulin. (C) Histological scores of colonic tissues in the acute DSS-induced colitis model. (D-F) Serum levels of pro-inflammatory cytokines, including TNF-α, IL-6, and IL-1β, in mice from the acute DSS-induced colitis model. (G-I) Relative mRNA expression levels of pro-inflammatory cytokines, including TNF-α, IL-6, and IL-1β, in colonic tissues from the acute DSS-induced colitis model. Data are presented as mean ± SEM. *P < 0.05, **P < 0.001, ***P < 0.0001; ns, not significant.

**Figure S6. F. nucleatum aggravates colitis severity and intestinal barrier injury across multiple experimental colitis models.**
 (A) Representative images of colon morphology in the chronic DSS-induced colitis model. (B) Quantification of colon length in the chronic DSS-induced colitis model. (C) Representative H&E-stained colon sections showing histopathological changes in the chronic DSS-induced colitis model. (D) Representative AB-PAS staining and MUC2 immunohistochemical staining showing goblet cells and mucin expression in the chronic DSS-induced colitis model. (E) Representative immunofluorescence staining of ZO-1 in colonic tissues from the chronic DSS-induced colitis model. (F) Representative images of colon morphology in the TNBS-induced colitis model. (G) Quantification of colon length in the TNBS-induced colitis model. (H) Representative H&E-stained colon sections showing histopathological changes in the TNBS-induced colitis model. (I) Representative AB-PAS staining and MUC2 immunohistochemical staining showing goblet cells and mucin expression in the TNBS-induced colitis model. (J) Representative immunofluorescence staining of ZO-1 in colonic tissues from the TNBS-induced colitis model. Data are presented as mean ± SEM. *P < 0.05, *****P*** < 0.01, ***P < 0.001, ****P < 0.0001; ns, not significant.

**Figure S7. F. nucleatum promotes pro-inflammatory macrophage activation in vivo and in vitro.**
(A) Representative flow cytometry plots showing Ly6C and MHCII expression in colonic lamina propria macrophages from PBS-, F. nucleatum-, DSS-, and F. nucleatum + DSS-treated mice.(B, C) Representative flow cytometry plots and quantification of CD86⁺CD206⁻ macrophages in the chronic DSS-induced colitis model.(D, E) Representative histogram and quantification of iNOS expression in intestinal macrophages from the chronic DSS-induced colitis model.(F, G) Representative histogram and quantification of TNF-α expression in intestinal macrophages from the chronic DSS-induced colitis model.(H, I) Representative histogram and quantification of Arg1 expression in intestinal macrophages from the chronic DSS-induced colitis model.(J) Representative immunofluorescence staining of F4/80 and iNOS in colonic tissues from the TNBS-induced colitis model. (K) Quantification of iNOS and Arg1 protein expression in BMDMs treated with PBS, F. nucleatum, LPS, or F. nucleatum + LPS. Protein levels were normalized to β-tubulin. (L-O) RT-qPCR analysis of pro-inflammatory mediators, including iNOS, TNF-α, IL-1β, and IL-6, in BMDMs from the indicated groups.(P) RT-qPCR analysis of CD206 expression in BMDMs from the indicated groups. Data are presented as mean ± SEM. *P < 0.05, *****P*** < 0.01, ***P < 0.001, ****P < 0.0001; ns, not significant.

**Figure S8. Heat-killed *F. nucleatum* fails to exacerbate DSS-induced colitis or promote pro-inflammatory macrophage activation.**

(A) Body weight changes in mice treated with PBS, heat-killed F. nucleatum, DSS, or heat-killed F. nucleatum + DSS. (B) Disease activity index (DAI) during DSS-induced colitis. (C) Representative images of colon morphology from the indicated groups. (D) Quantification of colon length. (E) Representative H&E-stained colon sections showing histopathological changes. (F) Histological scores of colonic tissues. (G) Representative flow cytometry plots showing CD86 and CD206 expression in colonic lamina propria macrophages. (H) Quantification of CD86⁺CD206⁻ macrophages in colonic lamina propria. Data are presented as mean ± SEM. ***P < 0.001, ****P < 0.0001; ns, not significant.

**Figure S9.** **Validation of SUCNR1 induction and siRNA-mediated SUCNR1 knockdown in BMDMs.**

(A) Quantification of SUCNR1 protein expression in BMDMs treated with PBS, F. nucleatum, LPS, or F. nucleatum + LPS. Protein levels were normalized to β-tubulin. (B) Quantification of SUCNR1 protein expression in BMDMs transfected with nontargeting siRNA (siNC) or three independent SUCNR1-targeting siRNAs (si1, si2, and si3). Protein levels were normalized to β-tubulin. (C) Representative Western blot analysis of SUCNR1 expression in BMDMs transfected with siNC, si1, si2, or si3. β-tubulin was used as the loading control. Data are presented as mean ± SEM. *P < 0.05, *****P*** < 0.01, ****P < 0.0001; ns, not significant.

**Figure S10. Succinic acid promotes pro-inflammatory macrophage activation and aggravates DSS-induced colitis.**
(A-C) RT-qPCR analysis of pro-inflammatory mediators, including **NOS2, IL-6,** and **TNF-α,** in BMDMs treated with control medium, succinic acid, LPS, or succinic acid + LPS. (D) RT-qPCR analysis of **CD206** expression in BMDMs from the indicated groups. (E) Representative immunofluorescence staining of iNOS in BMDMs. Nuclei were counterstained with DAPI. (F) Western blot analysis of iNOS, Arg-1, and SUCNR1 expression in BMDMs from the indicated groups. β-tubulin was used as the loading control. (G) Quantification of iNOS, Arg-1, and SUCNR1 protein expression normalized to β-tubulin. (H) Disease activity index (DAI) in DSS-treated mice with or without succinic acid supplementation. (I) Representative H&E-stained colon sections showing histopathological changes in the indicated groups. (J) Quantification of Western blot analysis showing the relative expression of ZO-1, occludin, Bcl-2, and Bax in colonic tissues. Protein levels were normalized to β-actin. (K) Representative immunofluorescence staining of ZO-1 in colonic tissues. Nuclei were counterstained with DAPI. (L-N) Serum levels of IL-6, TNF-α, and IL-1β in the indicated groups, as determined by ELISA. Data are presented as mean ± SEM. *P < 0.05, *****P*** < 0.01, ***P < 0.001, ****P < 0.0001; ns, not significant.

**Figure S11.** (A) Quantification of Western blot analysis showing iNOS, Arg-1, and SUCNR1 expression in BMDMs treated with LPS, Fn-WT + LPS, frdA-KO F. nucleatum + LPS, or frdA-KO F. nucleatum + LPS + succinic acid. Protein levels were normalized to β-tubulin. (B) Quantification of Western blot analysis showing ZO-1, occludin, Bcl-2, and Bax expression in colonic tissues from DSS-, Fn-WT + DSS-, frdA-KO F. nucleatum + DSS-, and frdA-KO F. nucleatum + DSS + succinic acid-treated mice. Protein levels were normalized to β-actin. (C–E) Serum levels of TNF-α, IL-6, and IL-1β in the indicated groups, as determined by ELISA. (F) Quantification of Western blot analysis showing p-p65/p65 and p-IκBα/IκBα ratios in BMDMs treated with control medium, LPS, succinic acid + LPS, Fn-WT + LPS, or frdA-KO F. nucleatum + LPS. (G) Quantification of Western blot analysis showing iNOS, Arg-1, p-p65/p65, p-IκBα/IκBα, and SUCNR1 expression in BMDMs treated with LPS, succinic acid + LPS, or Fn-WT + LPS in the presence of DMSO or the NF-κB inhibitor BAY 11-7082. Protein levels were normalized to β-actin where applicable. Data are presented as mean ± SEM. **P < 0.01, ***P < 0.001, ***P < 0.0001; ns, not significant.
